# Identification of Cross-Pathway Connections via Protein-Protein Interactions Linked to Altered States of Metabolic Enzymes in Cervical Cancer

**DOI:** 10.3389/fmed.2021.736495

**Published:** 2021-11-01

**Authors:** Krishna Kumar, Sarpita Bose, Saikat Chakrabarti

**Affiliations:** Structural Biology and Bioinformatics Division, Council of Scientific & Industrial Research (CSIR)-Indian Institute of Chemical Biology, Kolkata, India

**Keywords:** metabolic reprogramming, cervical cancer, mathematical modeling, systems biology, signaling pathway proteins, transcription factor, microRNA, metabolic enzymes

## Abstract

Metabolic reprogramming is one of the emerging hallmarks of cancer cells. Various factors, such as signaling proteins (S), miRNA, and transcription factors (TFs), may play important roles in altering the metabolic status in cancer cells by interacting with metabolic enzymes either directly or via protein-protein interactions (PPIs). Therefore, it is important to understand the coordination among these cellular pathways, which may provide better insight into the molecular mechanism behind metabolic adaptations in cancer cells. In this study, we have designed a cervical cancer-specific supra-interaction network where signaling pathway proteins, TFs, and microRNAs (miRs) are connected to metabolic enzymes via PPIs to investigate novel molecular targets and connections/links/paths regulating the metabolic enzymes. Using publicly available omics data and PPIs, we have developed a Hidden Markov Model (HMM)-based mathematical model yielding 94, 236, and 27 probable links/paths connecting signaling pathway proteins, TFs, and miRNAs to metabolic enzymes, respectively, out of which 83 paths connect to six common metabolic enzymes (RRM2, NDUFA11, ENO2, EZH2, AKR1C2, and TYMS). Signaling proteins (e.g., PPARD, BAD, GNB5, CHECK1, PAK2, PLK1, BRCA1, MAML3, and SPP1), TFs (e.g., KAT2B, ING1, MED1, ZEB1, AR, NCOA2, EGR1, TWIST1, E2F1, ID4, RBL1, ESR1, and HSF2), and miR (e.g., mir-147a, mir-593-5p, mir-138-5p, mir-16-5p, and mir-15b-5p) were found to regulate two key metabolic enzymes, EZH2 and AKR1C2, with altered metabolites (L-lysine and tetrahydrodeoxycorticosterone, THDOC) status in cervical cancer. We believe, the biology-based approach of our system will pave the way for future studies, which could be aimed toward identifying novel signaling, transcriptional, and post-transcriptional regulators of metabolic alterations in cervical cancer.

## Introduction

Cervical cancer is the fourth most frequently occurring cancer and the fourth leading cause of death in women worldwide with an estimate of 5,70,000 cases and 3,11,000 deaths in 2018. Approximately 80–85% of the deaths from cervical cancer occur in lower and middle-income countries compared to high-income countries (1, 2). Squamous cell carcinoma (SCC) and adenocarcinomas are the two main types of cervical cancer. Above 90% of patients with cervical cancer belong to SCC (3). The persistent infection with human papillomavirus (HPV), a particularly high-risk type of HPV (mainly HPV16 and HPV18 type), is considered the primary cause of cervical cancer (4–6). Only HPV16 and HPV18 types are responsible for almost 70% of cases of cervical cancer globally (7). While infection by high-risk HPV is necessary for developing cervical cancer, it alone may not be sufficient. Various studies suggest that the pathogenesis of cervical cancer depends on various other factors acting in concert with disease-associated HPV types (8–10). Therefore, it is important to understand the molecular mechanism behind the development of cervical cancer.

Metabolic reprogramming is considered one of the emerging hallmarks of cancer cells, and it is essential for cancer cell growth and proliferation to evolve into a more aggressive malignant state (11, 12). Understanding the coordination among various biological pathways, such as gene-regulatory, signaling, and metabolic pathways is important and may provide clues into the molecular mechanism of metabolic adaptation in cancer and associated cells. To understand that, one needs to investigate the molecular mechanism by which the impact of signaling, transcriptional, and post-transcriptional aberration is transgressed to metabolic reprogramming. Various studies demonstrated that the metabolic status in cancer cells is regulated by oncogenic changes in signaling pathways (13–15), transcription factors (TFs) (16–18), and miRNAs (19–21). However, these studies are focused either on a single molecule or pathways and may not capture the complex interconnectivity among various biological processes.

To overcome the complexity of interconnected biological pathways, biological approaches to efficient systems need to be developed. Computational and/or mathematical model-based system biology approaches provide an effective way to discover new drug targets for cancer therapy (22, 23). Mathematical model-based system biology approaches are successful for signaling and metabolic network analyses (24–30). Mathematical models for signaling pathways have been developed based on logical models (27–30), kinetic models (31, 32), Petri nets (33), decision tree (34), ordinary differential equations (35), and linear programming (LP)-based model (22, 36). Previously, we also have established a Hidden Markov Model (HMM)-based mathematical model to analyze the signaling-metabolic (S-M) interconnecting networks (37).

**Figure 1:**
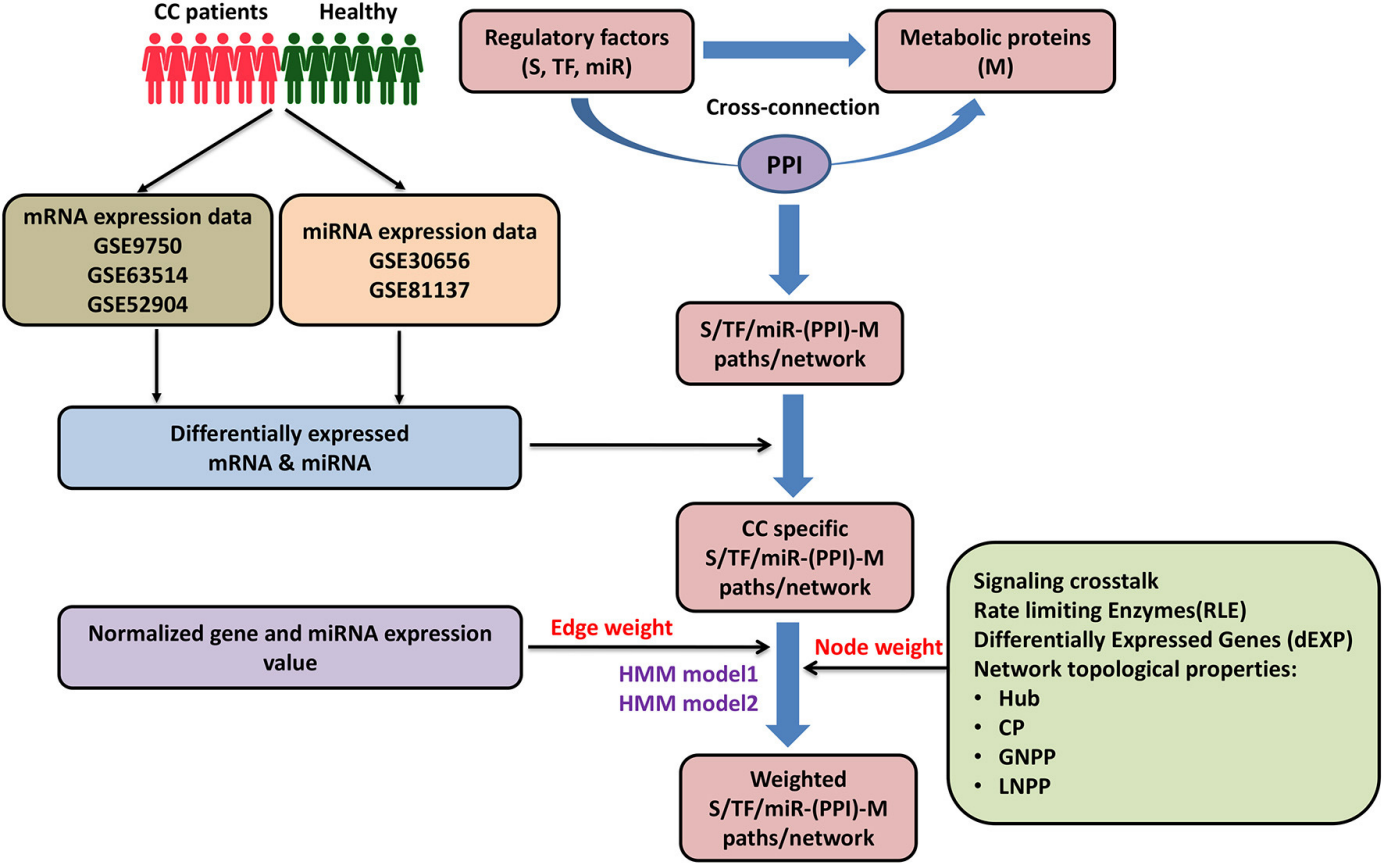
Pictorial workflow of the study. CC, cervical cancer; S, signaling proteins; TF, transcription factor; miR, microRNA; M, metabolic proteins; PPI, protein-protein interactors; CP, central proteins; LNPP, local network perturbing protein; GNPP, global network perturbing proteins.

In the present study, we have designed a cervical cancer-specific supra-interaction network model incorporating transcriptome data onto a protein-protein interaction (PPIs) network to investigate novel molecular targets and connections regulating the status of metabolic enzymes. We have developed a biology framework of a comprehensive system where signaling (S) pathway proteins, miRNA, and TF-based gene-regulatory modules are connected to metabolic (M) pathway proteins through protein-protein interactors (PPIs; Figure 1). Initially, network topologically IINs, such as a hub, central node (CN), local network perturbing nodes (LNPNs), and global network perturbing nodes (GNPN), were identified in different [S-M, TF-metabolic (TF-M), and miRNA-metabolic] modules of cervical cancer-specific networks using graph theory approach previously reported by our laboratory (38). These IINs may serve as potential diagnostic and/or prognostic biomarkers in cervical cancer. Furthermore, signaling pathway proteins, TFs, and microRNA (miR) to metabolic enzymes interconnecting paths/links [S-PPI-M, TF-target genes (TG)-PPI-M, and miR-TG-PPI-M] were identified in cervical cancer. Publicly available transcriptomic data derived from cervical cancer patients were incorporated into the HMM-based mathematical modeling set-up to weigh and rank the interconnecting link/paths in each module. Additional confidence values based on biological and network topological properties (hub, CN, GNPN, and LNPN) were assigned to each gene/protein/miRNA in the paths/links identified after model implementation to extract out high confident connections/links specific to cervical cancer. *In silico* validation of these selected genes/proteins/miRNAs and paths has been performed through perturbation analysis, demonstrating the importance of certain genes/proteins/miRNAs forming critical inter-pathway connections. PPI links connecting to key metabolic enzymes, such as RRM2, NDUFA11, ENO2, EZH2, AKR1C2, and TYMS, are identified from signaling proteins (e.g., PPARD, BAD, GNB5, CHECK1, PAK2, PLK1, BRCA1, MAML3, and SPP1), TFs (e.g., KAT2B, ING1, MED1, ZEB1, AR, NCOA2, EGR1, TWIST1, E2F1, ID4, RBL1, ESR1, and HSF2), and miR (e.g., mir-147a, mir-593-5p, mir-138-5p, mir-16-5p, and mir-15b-5p) in cervical cancer scenario. Out of the six metabolic enzymes that are commonly linked by 83 paths/links, EZH2 and AKR1C2 were mapped with deregulated metabolite status. Further, comparative analysis of the identified genes/proteins/miRNAs and the associated molecular pairs and paths in different modules were performed using transcriptomics data obtained from cervical, breast, and ovarian cancer patients. This study led to novel inter–bio-molecular links between signaling, gene-regulatory components, and metabolic enzymes paving the probable way(s) to identify drug targets to inhibit cervical cancer progression in a more specific manner.

## Materials and Methods

### Messenger Ribonucleic Acid (mRNA) and Micro Ribonucleic Acid (miRNA) Expression Datasets

mRNA and miRNA expression datasets of cervical, breast cancer, and ovarian cancer patients were extracted from the Gene Expression Omnibus (GEO) database (39) to study the gene and miRNA expression profiles. For the mRNA expression datasets, similar microarray (Affymetrix microarrays) platforms were used for each cancer type to minimize the undesirable variations that occurred due to different microarray platforms. Further, the cancer samples in each dataset were considered in a 1:1 ratio with normal samples to avoid sample heterogeneity (as shown in Table 1).

**Table 1:** Differential expression analysis of mRNA and miRNA.

**mRNA dataset**		**Origin**	**Normal** **samples**	**Patient samples**	**Up-Regulated**	**Down-Regulated**	**Neutrally expressed**
Cervical cancer	GSE9750	USA	19	19	330	634	4,338
	GSE63514	USA	19	19	1,019	676	4,390
	GSE52904	Mexico	17	17	177	322	6,228
	Total	–	45	45	1,250	1,331	10,053
Breast cancer	GSE29044	Saudi Arabia	27	27	356	729	9,175
	GSE42568	Ireland	17	17	1,270	1,343	7,636
	GSE103512	USA	10	10	57	101	8,016
	Total	–	54	54	1,385	1,676	1,2717
Ovarian cancer	GSE38666	USA	12	12	2,733	954	4,847
	GSE54388	USA	6	6	232	527	4,087
	GSE66957	USA	12	12	2,201	872	9,028
	Total	–	30	30	4,481	1,901	11,560
**miRNA dataset**		**Origin**	**Normal samples**	**Patient samples**	**Up-regulated**	**Down-regulated**	**Neutrally expressed**
Cervical cancer	GSE30656	Netherland	10	10	4	12	NA
	GSE81137	India	3	3	15	20	NA
	Total	–	13	13	19	31	NA
Breast cancer	GSE45498	Switzerland	59	59	66	13	NA
	GSE97811	Japan	16	16	39	15	NA
	GSE143564	China	3	3	141	20	NA
	Total	–	78	78	197	40	NA
Ovarian cancer	GSE23383	USA	3	3	53	15	NA
	GSE47841	Norway	9	9	52	105	NA
	GSE119056	China	3	3	55	147	NA
	Total	–	15	15	137	245	NA

### Differential Expression Analysis

The differential expression analysis of each dataset was performed separately using the GEO2R web tool (40–42) available at the GEO database. Genes having log_2_FC ≥ +1.5 and log_2_FC ≤ −1.5 were considered as upregulated and downregulated genes, respectively. The genes with log_2_FC values between −1.5 and +1.5 were considered only as neutrally expressed genes (EG). Benjamini and Hochberg's method (43) was used to control the false discovery rate. Genes with an adjusted *p* ≤ 0.05 were considered significant. For ovarian cancer datasets, the log_2_FC and *p*-value threshold of ± 2 and ≤ 0.01, respectively, were considered for the selection of upregulated, downregulated, and EG. For differential miRNA expression adjusted *p* ≤ 0.05 and log_2_FC ≥ +1.0 and log_2_FC ≤ −1.0 were considered as thresholds for the identification of upregulated and downregulated miRNAs, respectively.

### Construction of Human Protein-Protein Interaction Network

The HPPIN was constructed by extracting experimentally verified (confidence score ≥0.7) HPPIs available in the STRING v11.0 (44) database. The resulting network (proteins as nodes and edges demark interaction) consisted of 5,048 proteins and 18,044 interactions, respectively.

### Construction of Cancer-Specific Protein-Protein Interaction Network

Differentially expressed genes (dEXP) and EG from each cervical cancer dataset (GSE9750, GSE63514, and GSE52904; Table 1) were mapped onto the HPPIN to construct a cervical CC-PPIN. The interactions were considered up to the second level (i.e., interactors of interactors). In the first level, interactions mediated by only deregulated genes were considered where their interactors could be either deregulated or neutrally expressed. The resulting network consisted of 2,240 proteins interconnected via 5,452 edges. Similarly, breast and ovarian CC-PPINs were constructed using the corresponding transcriptomic datasets (Table 1).

### Construction of Cancer-Specific Transcriptional Regulatory Network

The transcriptional regulatory network was constructed by collating deregulated TF-TG interaction and associated protein-protein interactions. Experimentally verified strong evidenced TF-TG interactions were retrieved from Human Transcriptional Regulation Interactions database (HTRIdb) (45) and Transcriptional Regulatory Relationships Unraveled by Sentence-based Text mining (TRUSST) (46) databases and were merged generating 22,480 interactions among 697 TFs and 12,407 TG. The combined deregulated genes (differentially expressed and neutrally expressed) of all the three datasets (GSE9750, GSE63514, and GSE52904) were mapped on to TF-TG interactions to filter the cervical cancer-specific TF-TG interactions. In this study, only those interactions were considered where both TFs and their TG were deregulated. The deregulated TF-TG were further mapped to experimentally verify HPPIs up to the second level, and only those interactions were considered where the interacting partners were either deregulated or expressed. Finally, the filtered TF-TG and PPI interactions were merged to form the TF-TG-PPI network. The resulting network consisted of 2,894 nodes interconnected via 5,694 edges. Breast and ovarian cancer-specific TF-TG-PPI networks were also constructed with the corresponding mRNA transcriptomics data (Table 1) using the same protocol.

### Construction of Cancer-Specific Post-transcriptional Regulatory Network

The post-transcriptional regulatory network was constructed by collating miRNA-TG interactions and protein-protein interactions. The experimentally verified miRNA-TG interactions were retrieved from mirTarbase (47) and Tarbase (48) databases and merged, which consisted of 8,407 miRNA-TG interactions forming among 743 miRNA and 2,891 TG. Subsequently, the same procedure described for the construction of the TF-TG-PPIN network was followed for the construction of the miRNA-TG-PPIN network. The miRNA-TG-PPIN using cervical cancer-specific miRNA transcriptomics data consisted of 1,017 edges connecting 1,718 nodes. Likewise, breast and ovarian TF-TG-PPINs consisting of 2,668, 2,657 nodes and 6,197, 5,702 interactions, respectively, were also constructed.

### Characterization of Cancer-Specific Networks

The cancer-specific networks, PPIN, TF-TG-PPIN, and miRNA-TG-PPIN, described above were compared with the respective random networks of the same number of interactions. Ten random networks were generated by the NetworkX program (49) against each cervical cancer-specific network described above, and the degree distribution of each network was compared with the respective random networks. The degree distribution was calculated using the following formula:


$$P\left(k\right)=n_{k}/N$$

Where the degree distribution of network *P(k)* signifies the fraction of the node with degree *k*. For the network with a node size of N, *n*_*k*_ nodes will have the degree *k*.

### Identification of IINs

Topologically IINs (genes/proteins/miRNA) of the constructed cancer-specific networks described above were identified by utilizing procedures based on graph theory methods described earlier in Bhattacharyya and Chakrabarti (38). Identification of important interacting genes/proteins/miRNAs in the network is based on some independent network properties, such as hub (highly connected nodes in the network), CNs of the network, GNPN, and LNPN. The nodes (genes/proteins/miRNAs) that were identified as topologically important in at least two categories (Hub, CN, LNPN, and GNPN) were considered as IINs.

### Over-representation Analysis

Kyoto encyclopedia of genes and genomes (KEGG) pathway-based ORA was performed with deregulated genes extracted from the mRNA expression datasets used and IINs (except miRNAs) identified in each of the regulatory networks described above using “protein-coding gene set” as the reference gene set in WebGestalt (50) web tool. The top 20 pathway categories were ranked based on significant false detection rate (FDR) calculated using Benjamini and Hochberg procedure (43) and enrichment ratio.

Additionally, Gene Ontology (GO)-based molecular functions and online mendelian inheritance in man (OMIM)-based disease pathway over-representation analyses were also performed for the deregulated genes/proteins in cervical cancer.

### Construction of S-M Enzyme Cross-Connecting Paths and Network

A signaling-metabolic inter-connection network was constructed using 23 signaling pathway (cancer-specific) genes/proteins and all the metabolic pathways (85 pathways) genes/proteins. Signaling and metabolic gene/protein datasets were created by extracting all the genes/proteins from the KEGG (51, 52) database. All possible unique connections (maximum three proteins involved in between) to a metabolic pathway protein (M) were established through PPIs (up to the second level), considering a signaling pathway protein (S) as a starting point in the HPPIN. Four different types of linking paths were established where signaling proteins were connected to metabolic pathway proteins either directly (S-M) or via one (S-P-M), two (S-P-P-M), or three (S-P-P-P-M) PPIs, respectively. NetworkX program (49) was used to construct all possible signaling to metabolic interconnecting paths. These paths/connections were converted into a network to construct a signaling-metabolic interaction network (SMIN).

### Construction of TF to Metabolic Enzyme Cross-Connecting Paths and Network

Connections between TFs and metabolic pathway genes/proteins were established through TF-TG interactions and PPIs of TF-TG (up to the second level) considering TFs as a source. In this study, five different types of the path were established where TFs were connected to metabolic pathway proteins either directly (TF-TG/M), or TFs were connected to their TG, and their TG were connected to metabolic proteins directly (TF-TG-M), or through one (TF-TG-P-M), two (TF-TG-P-P-M), and three (TF-TG-P-P-P-M) PPIs of TG, respectively. The resulting paths/links were converted into a network to construct a TF-metabolic interaction network (TFMIN).

### Construction of miRNA to Metabolic Enzyme Cross-Connecting Paths and Network

MicroRNAs to metabolic pathway proteins interconnecting all possible paths were established using the NetworkX program (49). The miRs to metabolic pathway proteins (M) interconnecting paths were established using miRNA-TG interactions and PPIs (up to the second level) of miRNA TG considering miRNA as the source. The resulting paths were of five types viz; miRNA-TG/M, miR-TG-M, miR-TG/P-P-M, miR-TG/P-P-P-M, and miR-TG/P-P-P-P-M paths. The resulting paths were converted into a network to form a miR-metabolic interconnecting network (miRMIN).

### Contextualization of Regulatory Molecules (Signaling Pathway Proteins, TF, and miRNA) to Metabolic Enzyme Cross-Connecting Paths and Network

The deregulated (upregulated and downregulated) and neutrally EG and miRNAs identified from the cervical, breast, and ovarian cancer patients specific transcriptomic datasets were mapped onto all possible paths/connections mentioned above to filter cancer-specific regulatory molecules (signaling pathway proteins, TF, and miRNA) to metabolic enzymes cross-connecting paths. For the signaling to metabolic interconnecting paths/connections/links, the paths having deregulated (upregulated and downregulated) genes/proteins at the terminals and deregulated or EG/proteins in middle were filtered out and converted into a network to form cervical, breast, and ovarian cancer-specific signaling-metabolic interconnecting subnetwork (CC-SMIN, BC-SMIN, and OC-SMIN, respectively).

Similarly, to construct the cancer-specific TF and miRNA to metabolic interconnecting sub-networks (CC/BC/OC-TFMIN and CC/BC/OC-miRMIN, respectively), the paths having deregulated genes/miRNA at the terminal and their target and deregulated or EG/proteins in the middle were considered. The respective resulting paths were converted into a network to form cancer-specific (CC/BC/OC-TFMIN and CC/BC/OC-miRMIN) sub-networks.

### Calculation of Edge Weight

The edge weights in each sub-networks (CC/BC/OC-SMIN, CC/BC/OC-TFMIN, and CC/BC/OC-miRMIN) were defined in terms of local entropy using an in-house program (37). The probability of interactions of a gene/protein with its interactors in the sub-network was determined by using the principle of mass action to define the local entropy of a gene/protein. The calculation of the interaction probabilities is based on the assumption that two proteins known to interact will have a higher probability of interaction when they are highly expressed. The normalized expression values of sub-network genes in the samples of cancer patients used in this study were utilized to calculate the interaction probabilities.

### Calculation of Node Weight and Effect-On-Node

To incorporate the importance and impact of the interactors of a particular node in the sub-networks, the node-weight (*W*_*i*_) of every node *i* was defined based on its biological properties [signaling cross-talk (SC) protein and rate-limiting enzyme (RLE)], differentially expressed gene (dEXP), and network topological properties [hub, CP, local network perturbing protein (LNPP), and global network perturbing proteins (GNPP)] using the following formula.


$$W_{i}=\;\{\begin{array}{c} 1;if\;(dEXP=\frac{1}{7},\;RLE=\;\frac{1}{7},\;SC=\;\frac{1}{7},\;HUB=\;\frac{1}{7},\\ CP=\frac{1}{7},\;GNPP=\;\frac{1}{7},\;LNPP=\;\frac{1}{7})\\ 0;else \end{array}$$

Effect of interactors on a node in the sub-network was defined as effect-on-node (*effs*) depending on the node weight of its interactors up to the second level.


$$effs=\;\sum_{j}^{k}\left(\sum_{i}^{n}\frac{w_{i}}{n_{i}}+\;\frac{w_{j}}{n_{j}}\right)$$

Where *k* is the degree of node *s*, *n*_*i*_ is the degree of node *i*, *n*_*j*_ is the degree of node *j*, *and w*_*i*_ and *w*_*j*_ are the weights of nodes *i* and *j*.

### Identification of Significant Regulatory Molecules (S/TF/miR) to a Metabolic Enzyme (M) Interconnecting Pairs and Path

To understand the information flow starting from a regulatory molecule (S/TF/miRNA) to metabolic enzyme (M), cancer-specific cross-connecting paths mentioned above were scored by implementing an HMM-based mathematical model established in our laboratory earlier (37). In this study, two separate models were used to identify the significant S/TF/miR–M pairs (the source; signaling pathway protein/TFs/miR; and destination: metabolic pathway protein) and S/TF/miR–M interconnecting paths. Model 1 was applied to identify the S/TF/miR–M pairs. Model 2 was applied to identify the S/TF/miR–M interconnecting paths between the S/TF/miR–M pairs selected after Model 1. Edge weight and node weight of genes/proteins/miRNAs involved in the S/TF/miR–M path were used to calculate the path scores. The path score of each S/TF/miR to M linking path calculated by Model 1 and Model 2 was converted into a statistical z-score to identify paths deviating from the mean. A z-score (Z) ≥ 1 filter was applied to select the significant S/TF/miR–M pairs. Paths having path score ≥ 80% of the highest path score for every S/TF/miR–M pair were considered as significant S/TF/miR–M interconnecting paths from Model 2.

All the networks were visualized and represented by using Cytoscape (53). The signaling, TF, and miR to metabolic pathway connections were represented as the Circos plot (54).

### *In-silico* Perturbation Analysis

*In-silico* perturbation analysis was performed for each signaling pathway protein, TF, and miRNA-based gene regulatory module to identify the paths that change significantly upon removal of a node (protein/TF/miRNA). To identify the key nodes in the final paths/networks of every module, each of the nodes present in the paths/network having z-score ≥ 1 was removed individually from the HPPIN, and the path score was recalculated for the resulting paths/network by using the HMM Models 1 and 2. The perturbation score was calculated by using the average path score before and after perturbation as below.


$$Perturbation_{score}=\;Path_{score^{\prime }}-\;Path_{score}$$

Where, *Path*_*score*_ and $Path_{score^{\prime }}$ are average path scores before and after perturbation, respectively.

The difference of average path score (before vs. after perturbation) for each perturbed node was converted into a z-score (Z), and the nodes for which z-scores deviated from the mean as −1 ≥ *Z* ≥ 1 were selected as effective or key nodes in significant paths/network.

### Metabolomics Data Collection and Integration Into Cancer-Specific Paths

The deregulated metabolites in cervical, breast, and ovarian cancer patient were extracted from literature (55–57). The cervical cancer metabolomic dataset consisted of 55 downregulated and seven upregulated metabolites. Twenty-one downregulated and 41 upregulated metabolites were found in breast cancer whereas the ovarian cancer metabolomic dataset consisted of 46 downregulated and 116 upregulated metabolites. The metabolic genes corresponding to these metabolites were obtained from the Human metabolome database (HMDB) (58). The deregulated metabolites were mapped to the paths obtained after model implementation.

### Survival Analysis

Kaplan-Meier (KM) plotter software (59, 60) was used to perform the overall survival (OS) analysis of the constituent genes/miRNAs of the identified cross-pathway paths/links. We used a KM plotter using survival and expression data of 307 cervical cancer patients obtained from the TCGA dataset (project ID: TCGA-CESC; phs000178). To estimate the survival prognostic value of a specific gene/miRNA, the patient samples were divided into high- and low-expression cohorts according to the median expression of the given gene/miRNA, and KM plots were created. Additionally, the hazard ratio (HR) and the log-rank *p*-value were calculated. The survival estimate of a gene/miRNA with a *p*-value < 0.05 was considered to be statistically significant.

### Drug/Chemotherapy Response Analysis

The receiver operator characteristic (ROC) plotter (61) was used to predict the utility of the genes as predictive biomarkers with respect to drug/chemotherapy response. ROC plotter is capable to link gene expression and response to therapy using transcriptome-level data of 3,104 breast cancer patients.

## Results

### mRNA and miRNA Expressions in Cervical Cancer Patients

**Figure 2:**
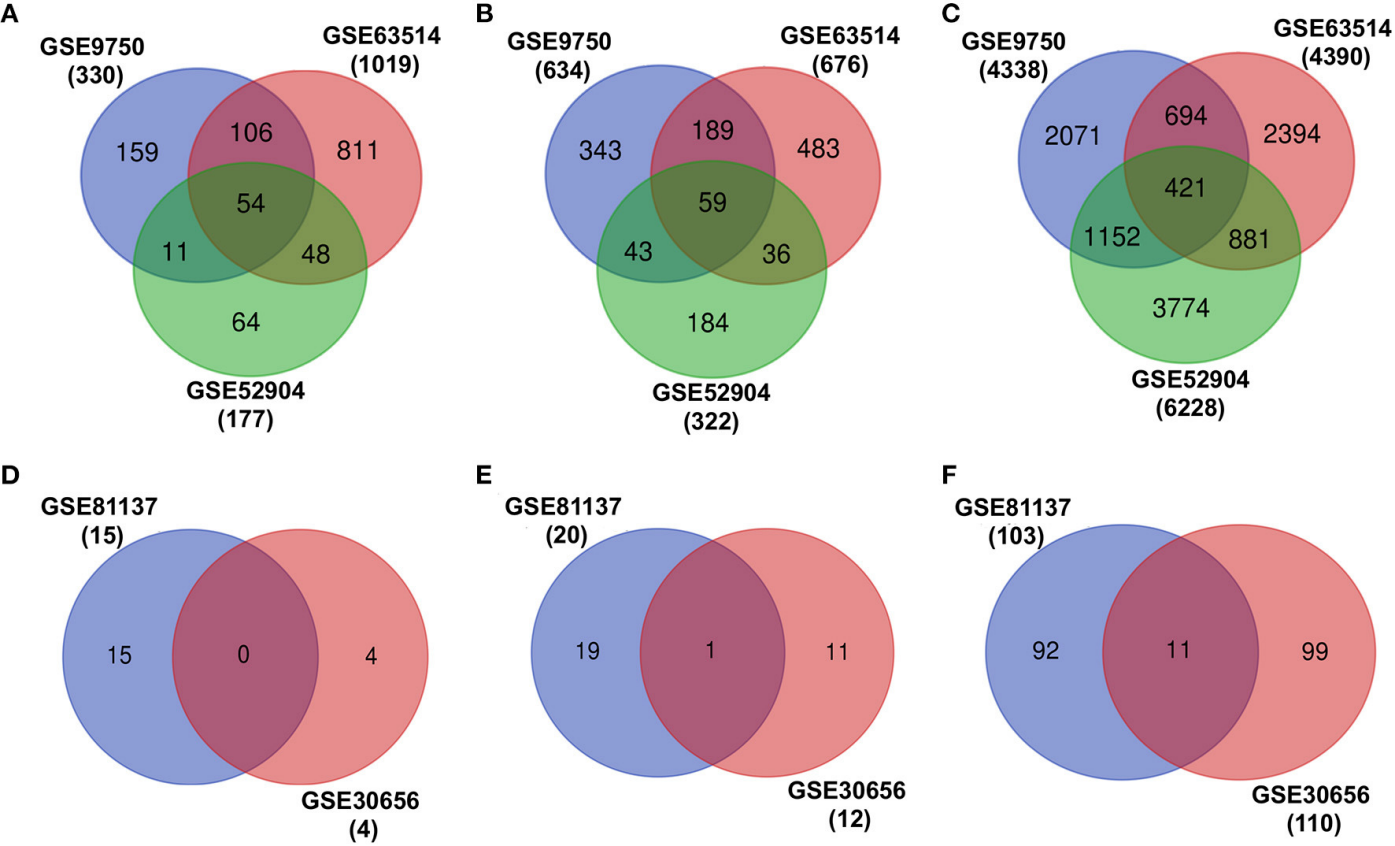
Differential expression analysis of transcriptomic datasets associated with cervical cancer. **(A–C)** represent the overlap of upregulated, downregulated, and EG, respectively. **(D–F)** show the overlap of upregulated, downregulated, and expressed miRNAs, respectively.

The individual differential expression analysis of three different cervical cancer mRNA expression datasets (GSE9752, GSE63514, and GSE52904) leads to the identification of several upregulated, downregulated, and neutrally EG in cervical cancer (Figure 2, Table 1). However, a comparison of the gene expression patterns across datasets showed that only 54 upregulated, 59 downregulated, and 421 neutrally EG were found to be overlapped (Figures 2A–C). The differential expression analysis of miRNA resulted in four upregulated, 12 downregulated, and 110 neutrally expressed miRNAs in GSE30656 and 15 upregulated, 20 downregulated, and 103 neutrally expressed miRNAs in the GSE81137 dataset (Figures 2D–F).

Over-representation analysis-based enrichment for cellular pathways, molecular functions, and biological processes was performed using a merged list of deregulated (upregulated and downregulated) genes of all the three mRNA datasets. Supplementary Figures 1A,B show the top 20 most enriched pathway filters based on FDR for proteins encoded by upregulated and downregulated genes, respectively. Most highly enriched pathways were found to be DNA replication (*p* = 4.42E^−13^) and arachidonic acid metabolism (*p* = 2.76E^−08^) for the proteins encoded by upregulated and downregulated genes, respectively. GO-based molecular function and biological process ORA was also performed using deregulated genes in cervical cancer. The most significantly enriched molecular functions were found to be collagen binding (*p* = 4.36E^−07^) and oxidoreductase activity (*p* = 4.35E^−04^) for proteins encoded by upregulated and downregulated genes, respectively. However, microtubule cytoskeleton organization involved in mitosis (*p* = 2.13E^−14^) and peptide cross-linking (*p* = 8.81E^−11^) were highly enriched biological processes, for upregulated gene-encoded proteins and downregulated gene-encoded proteins, respectively (Supplementary Figure 1).

### Construction and Characterization of Cervical Cancer-Specific Networks

The cervical CC-PPIN, TF-TG-PPIN, and miR-TG-PPIN were constructed by mapping differentially expressed genes, miRNA, and neutrally EG from each cervical cancer dataset (see Methods; Supplementary Figures 2A, 3A, 4A, respectively). The resulting CC-PPIN, TF-TG-PPIN, and miR-TG-PPIN were validated by comparing them with corresponding 10 random networks of the same size. The degree distributions of CC-PPIN, TF-TG-PPIN, and miR-TG-PPIN networks followed the Power law and were considered to have scale-free organization. However, the degree distribution of 10 corresponding random networks showed binomial distribution (Supplementary Figures 2B, 3B, 4B).

### IINs in Cervical Cancer-Specific Networks

Network topologically important nodes, such as hubs (highly connected nodes in the network), CNs, LNPNs, and GNPNs in a scale-free network could play important roles in maintaining the network integrity and function. Various topologically important interacting genes/proteins/miRNAs (hubs, CN, GNPN, and LNPN) in cervical cancer-specific networks (CC-PPIN, TF-TG-PPIN, and miR-TG-PPIN) were identified by implementing a graph theory-based method described earlier by Bhattacharyya and Chakrabarti (38). A total of 165, 167, 96, and 67 nodes/proteins in CC-PPIN (Supplementary Figure 2C, Supplementary Table 1), 62, 36, 60, and 80 nodes/genes/proteins in TF-TG-PPIN (Supplementary Figure 3C, Supplementary Table 2), and 45, 45, 30, and 24 nodes/genes/proteins/miRNAs in miR-TG-PPIN (Supplementary Figure 4C, Supplementary Table 3) were identified as hubs, CNs, GNPNs, and LNPNs, respectively. The nodes/genes/proteins/miRNAs common in at least any two of the four categories (hubs, CN, GNPN, and LNPN) were considered as IINs in cervical cancer networks. When the IINs from each network were compared, 30 IINs were found to be common in CC-PPIN and TF-TG-PPIN, 38 IINs were common in CC-PPIN, and miR-TG-PPIN, and 17 IINs were shared by TF-TG-PPIN and miR-TG-PPIN. However, 17 IINs were found to be common in all three cervical cancer networks (Supplementary Figure 5A, Supplementary Table 4).

Over-representation analysis-based pathway enrichment was performed using IINs in the above described cervical cancer-specific regulatory networks. Top three enriched pathways were found to be DNA replication (*p* = 1.30E^−10^), basal TFs (*p* = 3.07E^−10^), and cell cycle (*p* = 0.00) for IINs in CC-PPIN (Supplementary Figure 2D). DNA replication (*p* = 1.21E^−10^), mismatch repair (*p* = 1.29E^−04^), and cell cycle (*p* = 1.44E^−15^) were the top three enriched pathways for IINs in TF-TG-PPIN (Supplementary Figure 3D), respectively. However, for IINs (except miRNA) of miR-TG-PPIN, top three enriched pathways were found to be DNA replication (*p* = 8.88E^−16^), cell cycle (*p* = 0.00), and mismatch repair (*p* = 5.23E^−06^; Supplementary Figure 4D). When the top 20 enriched pathways for IINs in each network were compared, 8 enriched pathways were found to be common (Supplementary Figure 5B). The common pathways were cell cycle, DNA replication, nucleotide excision repair, mismatch repair, prostate cancer, herpes simplex infection, oocyte meiosis, and viral carcinogenesis pathways.

### S-M Enzyme Cross-Connecting Paths and Network in Cervical Cancer

Experimentally supported HPPIs (experimental score ≥ 0.7) were utilized to establish all possible S-M cross-connecting paths, where signaling pathway proteins were connected to metabolic enzymes either directly (S-M paths) or via one (S-P-M paths), two (S-P-P-M paths), and three (S-P-P-P-M paths) PPIs in between them. The resulting S-M cross-connecting paths consisted of 210 direct (S-M) connections, 2,669 via one PPI (S-P-M), 40,266 via two PPIs (S-P-P-M), and 7,35,395 via three PPIs (S-P-P-P-M) interconnections. These interconnections/paths were formed between 210, 1,697, 7,965, and 28,920 S-M pathway protein pairs, respectively. These S-M paths were converted into a network to form a SMIN, which consisted of 11,442 interactions formed among 2,603 genes/proteins.

To understand the flow of information from a signaling protein to metabolic enzymes probably leading to metabolic adaptations in the case of cervical cancer cells, we mapped differentially expressed (up and downregulated) and neutrally EG onto the abovementioned S-M interconnecting paths. The S-M paths having deregulated genes at the terminal and deregulated or EG in middle were extracted and considered for further analysis. The resulting paths/interconnections consisted of four (S-M), 47 (S-P-M), 639 (S-P-P-M), and 10,311 (S-P-P-P-M) paths formed between 4, 39, 180, and 631 S-M pairs, respectively. The filtered paths were converted into a network to construct the cervical cancer-specific SMIN (CC-SMIN) network. The CC-SMIN consisted of 1,425 interactions forming among 439 genes/proteins.

**Figure 3:**
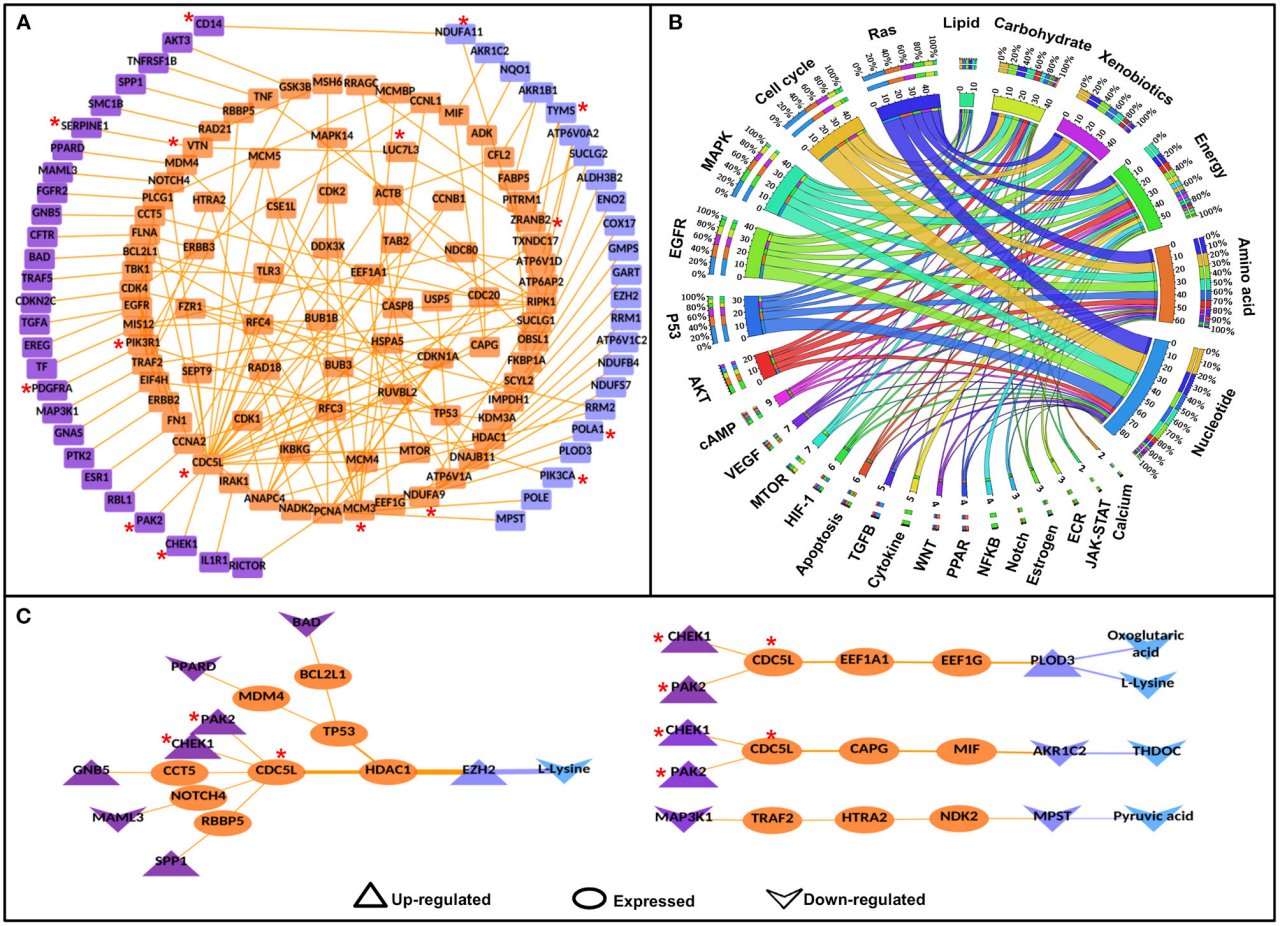
Significant signaling-metabolic pathway interconnecting paths/network in cervical cancer. **(A)** represents a significant signaling metabolic interaction network, **(B)** shows the signaling metabolic pathways interconnectivity, and **(C)** shows the significant signaling to metabolic paths regulating metabolites in cervical cancer. Terminal signaling pathway proteins and metabolic enzymes are colored in purple and blue. Protein-protein interactors are colored in orange. Protein-protein interactions are represented by orange edges. Nodes with an asterisk (*) are key or effector nodes in the significant paths/network.

To identify the potential disease-specific paths/pairs, each node and each edge of the CC-PPIN network were weighted based on their biological properties, differential expression status, and network topological properties (hubs, CN, GNPN, and LNPN) of CC-PPIN. Local signaling entropy (S_i_) was integrated to understand the system-level network property. The significance of each node (gene/protein) in the cancer-specific network was estimated in the form of effect-on-node (*effs*) based on SC, RLE, dEXP (differentially expressed genes), hub, CN, GNPN, and LNPN, respectively. To identify the probable and significant paths of information flow from signaling pathway to metabolic pathway in cervical cancer cells, local signaling entropy (S_i_) and effect-on-node (*effs*) properties were incorporated as node weights. The edge weight of every two interacting nodes of CC-PPIN was defined as the probability of interaction using their normalized expression value in cervical cancer patient samples. Node weight and edge weight were integrated into HMM-based mathematical models (Models 1 and 2) to identify S-M linking pairs and paths. Model 1 was applied to identify the S-M pairs (the source signaling pathway protein and destination metabolic pathway protein). Model 2 was applied to identify the S-M interconnecting paths between the S-M pairs selected after Model 1. The path score of each S-M linking path calculated by Models 1 and 2 was converted into a statistical z-score to identify paths deviating from the mean. A z-score ≥ 1 filter was applied to select the significant S-M pairs. Using these filtering criteria, we identified 81 S-M pairs and 94 S-PPI-M paths in cervical cancer. The selected paths were converted into a network to form a significant CC-PPIN network which consisted of 152 interactions forming among 135 genes/proteins (Table 2, Figure 3A).

**Table 2:** Signaling to metabolic pathways interconnecting paths and pairs.

**Connection types**	**Pairs and paths**							
	**Signaling-Metabolic interaction network (SMIN)**		**Cervical cancer (CC)**		**Breast cancer (BC)**		**Ovarian cancer (OC)**	
			**Significant CC-SMIN**		**Significant BC-SMIN**		**Significant OC-SMIN**	
			**Model 1** **pair selection**	**Model 2** **paths selection**	**Model 1** **pair selection**	**Model 2** **paths selection**	**Model 1** **pair selection**	**Model 2** **paths selection**
	**Pairs**	**Paths**	***Z* ≥ 1** **pairs**	***Z* ≥ 1 or** **score ≥ 80%** **paths**	***Z* ≥ 1** **pairs**	***Z* ≥ 1 or** **score ≥ 80% paths**	***Z* ≥ 1** **pairs**	***Z* ≥ 1 or** **score ≥ 80%** **paths**
S-M	210	210	1	1	1	1	2	2
S-P-M	1,697	2,669	1	1	9	12	7	9
S-P-P-M	7,964	40,266	15	14	57	79	39	48
S-P-P-P-M	28,920	735,395	79	78	223	187	193	191
Total	29,179	778,540	81	94	232	279	218	250

*S-M, signaling-metabolic; S-P-M, single protein-protein interaction; S-P-P-M, two protein-protein interactions; S-P-P-P-M, three protein-protein interactions*.

Mapping pathway information to the terminal nodes (source signaling protein and destination metabolic enzyme) showed that the Ras signaling pathway had maximum connections (50) to all the six metabolic pathways followed by cell cycle (44), Map-kinase (44), epidermal growth factor receptor (EGFR) signaling pathway (42), and p53 signaling pathway (34), respectively. However, among metabolic pathways, nucleotide metabolism had maximum connections (87) with signaling pathways, followed by amino acid (63), energy (58), xenobiotics (44), carbohydrate (42), and lipid metabolism (11), respectively. 1:1 interconnections between signaling and metabolic pathways showed that the cell cycle had maximum connections with nucleotide metabolism (18 connections; Figure 3B, Supplementary Table 5).

Mapping the deregulated metabolites in cervical cancer to significant S-M paths yielded 12 S-PPI-M interconnections/paths where four metabolites [L-lysine, oxoglutaric acid, tetrahydrodeoxycorticosterone (THDOC), and pyruvic acid] were regulated by eight signaling pathway proteins (BAD, CHEK1, GNB5, MAML3, MAP3K1, PAK2, PPARD, and SPP1). The metabolic enzymes connecting these four metabolites were enhancers of zeste homolog 2 (EZH2), procollagen lysine hydroxylase and glycosyltransferase LH3 (PLOD3), aldo-keto reductase family 1 member C2 (AKR1C2), and 3-mercaptopyruvate sulfurtransferase (MPST). L-lysine is the substrate of both EZH2 and PLOD3. Whereas, THDOC and pyruvic acid are the products of metabolic enzymes AKR1C2 and MPST, respectively. Hence, these paths/connections showed the correlated status of the metabolic enzymes and the corresponding metabolites (Figure 3C).

### TF to Metabolic Enzyme Cross-Connecting Paths and Network in Cervical Cancer

All possible paths/links connecting TF to the metabolic enzyme (M) were established using TF-TG interaction and HPPIN (refer to Methods). The resulting paths/links consisted of 930 TF-TG/M paths, 4,276 TF-TG/P-M, 114,844 TF-TG/P-P-M, 9,384,069 TF-TG/P-P-P-M, and 188,563,171 TF-TG/P-P-P-P-M paths forming between 930, 2,299, 9,121, 33,676 and 90,477 TF-M pairs, respectively. These paths were converted into a network to form a TFMIN.

All possible TF-TG-PPI-M paths were filtered by mapping deregulated and neutrally EG to establish the context-specific (cervical cancer-specific) paths and network. The resulting paths consisted of 89 TF-TG/M, 20 TF-TG/P-M, 110 TF-TG/P-P-M, 1,262 TF-TG/P-P-P-M, and 21,016 TF-TG/P-P-P-P-M paths formed between 89, 17, 53, 162 and 508 TF-M pairs, respectively. The filtered paths were converted into a network to construct the cervical cancer-specific TFMIN (CC-TFMIN), which consisted of 815 nodes and 2,364 edges formed among them.

**Figure 4:**
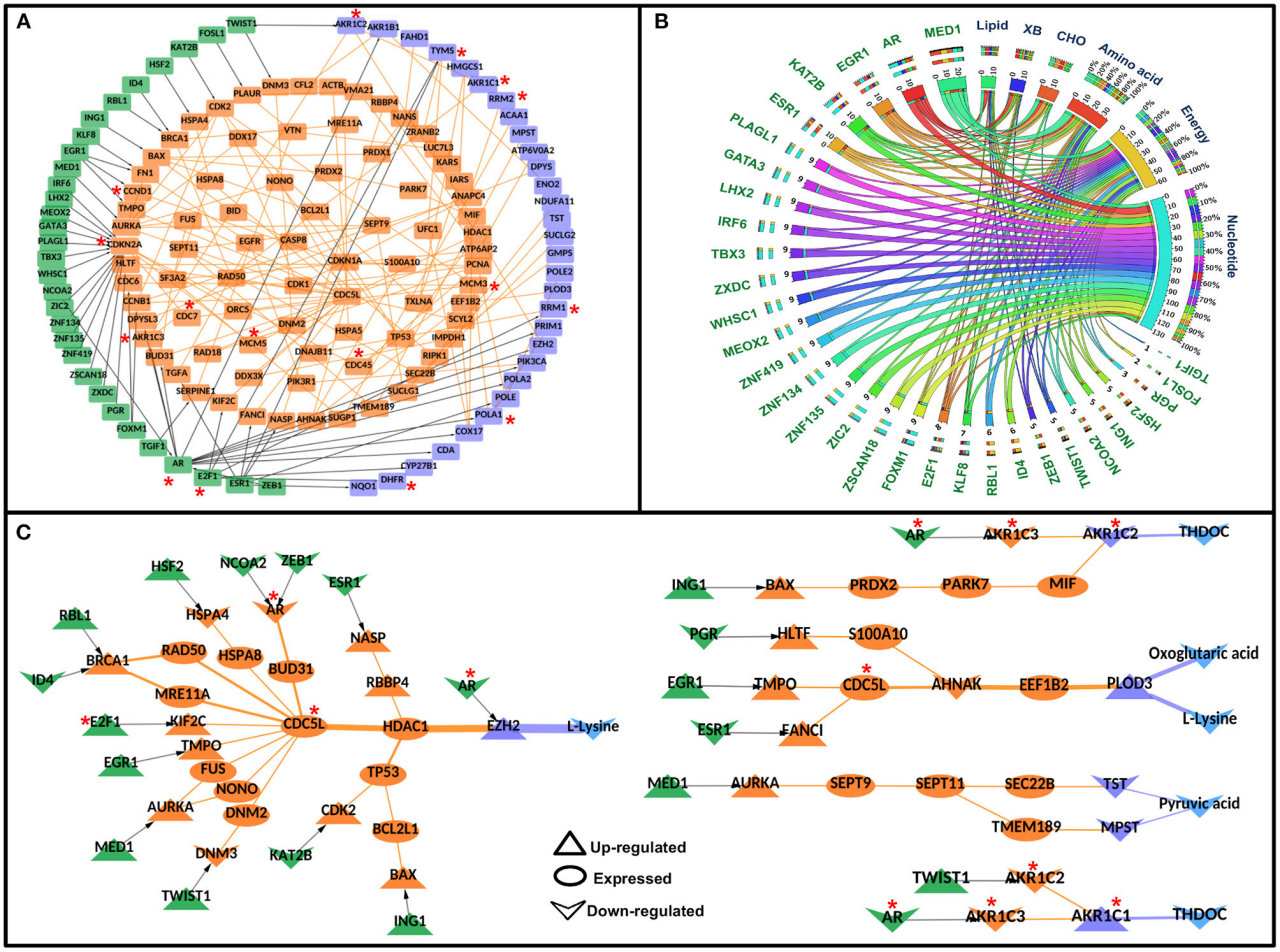
Significant Transcription factor-metabolic pathway interconnecting paths/network in cervical cancer. **(A)** represents a significant transcription factor metabolic interaction network, **(B)** shows the transcription factor to metabolic pathways interconnectivity, and **(C)** shows the significant transcription factor to metabolic paths regulating metabolites in cervical cancer. Terminal transcription factors and metabolic enzymes are colored green and blue. Protein-protein interactors are colored in orange. Gene regulatory edges are represented as black arrows and protein-protein interactions are represented by orange edges. Nodes with an asterisk (*) are key or effector nodes in the significant paths/network.

Each node and each edge in the CC-TFMIN were weighted to identify the potential significant paths in the cervical cancer-specific network. Node weights and edge weights were incorporated into HMM-based mathematical models (Models 1 and 2) to identify TF-M pairs and TF-PPI-M paths forming between them (refer to Methods). Model 1 resulted in the identification of 172 significant (*z* ≥ 1) TF-M pairs and Model 2 resulted in 236 significant TF-PPI-M paths connecting the TF-M pairs obtained after Model 1. The significant TF-PPI-M paths in cervical cancer were converted into a network to form significant CC-TFMIN that consisted of 179 interactions among 141 genes/proteins (Figure 4A, Table 3).

**Table 3:** Transcription factor to metabolic pathways interconnecting paths and pairs.

**Connection types**	**Pairs and paths**							
	**TF-Metabolic interaction network (TFMIN)**		**Cervical cancer (CC)**		**Breast cancer (BC)**		**Ovarian cancer (OC)**	
			**Significant CC-TFMIN**		**Significant BC-TFMIN**		**Significant OC-TFMIN**	
			**Model 1** **pair selection**	**Model 2** **paths selection**	**Model 1** **pair selection**	**Model 2** **paths selection**	**Model 1** **pair selection**	**Model 2** **paths selection**
	**Pairs**	**Paths**	***Z* ≥ 1** **Pairs**	***Z* ≥ 1 or** **score ≥ 80% paths**	***Z* ≥ 1** **pairs**	***Z* ≥ 1 or** **score ≥ 80% paths**	***Z* ≥ 1** **pairs**	***Z* ≥ 1 or** **score ≥ 80% paths**
TF-TG/M	930	930	6	14	2	2	2	3
TF-TG/P-M	2,299	4,276	5	6	3	4	5	12
TF-TG/P-P-M	9,121	114,844	10	9	11	18	4	17
TF-TG/P-P-P-M	33,676	9,384,069	53	66	46	87	116	155
TF-TG/P-P-P-P-M	90,477	188,563,171	159	141	251	200	562	482
Total	91,790	193,067,290	172	236	261	311	577	669

*TF-TG/M, TFs connected to metabolic pathway proteins; TF-TG-M, TFs connected to TG and TG connected to metabolic proteins directly; TF-TG-P-M, TFs connected to TG and TG connected to metabolic proteins directly through one PPI of TG; TF-TG-P-P-M, TFs connected to TG and TG connected to metabolic proteins directly two PPIs of TG; TF-TG-P-P-P-M, TFs connected to TG and TG connected to metabolic proteins directly three PPIs of TG*.

The metabolic pathway information was mapped onto the terminal metabolic enzymes of the significant TF-PPI-M paths to identify metabolic pathways that are highly connected to specific TFs. Nucleotide metabolism yielded maximum connections followed by energy metabolism, amino acid metabolism, carbohydrate metabolism, lipid metabolism, and xenobiotics biodegradation and metabolism (Figure 4B, Supplementary Table 6).

Mapping of deregulated metabolites onto the terminal metabolic enzymes resulted in 28 paths where four metabolites were deregulated in cervical cancer. The deregulated metabolites were L-lysine, oxoglutaric acid, pyruvic acid, and THDOC. L-lysine was found to be linked with AR, ESR1, ZEB1, NCOA2, HSF2, RBL1, ID4, E2F1, EGR1, MED1, TWIST1, KAT2B, ING1, and PGR TFs via 19 different paths. Oxoglutaric acid was linked with PGR, EGR1, and ESR1 via three paths. MED1 was found to be regulating (probably) pyruvic acid whereas THDOC was found to be linked with AR, TWIST1, and ING1 in four different paths (Figure 4C).

### miR to Metabolic Enzyme Cross-Connecting Paths and Network in Cervical Cancer

Similar to SMIN and TFMIN, all possible paths/links were established considering miRNA as a source node and metabolic enzymes as a destination by collating miRNA-TG interactions and HPPIN. The resulted paths/links consisted of 577 direct (miR-TG/M) paths, 1,145 via their TG (miR-TG/P-M), 26,330 via one PPI (miR-TG/P-P-M), 826,207 via two PPI (miR-TG/P-P-P-M), and 33,934,931 via three PPI (miR-TG/P-P-P-P-M) paths formed by 577, 1,128, 9,904, 44,271, and 111,730 miR-M pairs, respectively. The deregulated miRNA, genes, and neutrally EG were mapped onto all possible miR-PPI-M paths to filter the cervical cancer-specific miRNA to metabolic enzymes interconnections. The filtered paths/links consisted of 13 miR-TG/M, 1 miR-TG/P-M, 7 miR-TG/P-P-M, 95 miR-TG/P-P-P-M, and 1,851 miR-TG/P-P-P-P-M paths formed between 13, 1, 7, 38 and 149 miR-M pairs, respectively. The resulted paths were converted into a network to form a cervical cancer-specific miR-metabolic enzyme interaction network (CC-miRMIN) which consisted of 309 nodes and 952 interactions among them (Table 4).

**Table 4:** microRNA to metabolic pathways interconnecting paths and pairs.

**Connection types**	**Pairs and paths**							
	**miRNA-Metabolic interaction network (miRMIN)**		**Cervical cancer (CC)**		**Breast cancer (BC)**		**Ovarian cancer (OC)**	
			**Significant CC-miRMIN**		**Significant BC-miRMIN**		**Significant OC-miRMIN**	
			**Model 1** **pair selection**	**Model 2** **paths selection**	**Model 1** **pair selection**	**Model 2** **paths selection**	**Model 1** **pair selection**	**Model 2** **paths selection**
	**Pairs**	**Paths**	***Z* ≥ 1** **pairs**	***Z* ≥ 1 or** **score ≥ 80% paths**	***Z* ≥ 1** **pairs**	***Z* ≥ 1 or** **score ≥ 80% paths**	***Z* ≥ 1** **pairs**	***Z* ≥ 1 or** **score ≥ 80% paths**
miR-TG/M	577	577	1	2	9	9	0	0
miR-TG/P-M	1,128	1,145	1	1	4	6	2	2
miR-TG/P-P-M	9,904	26,330	2	2	14	34	5	8
miR-TG/P-P-P-M	44,271	826,207	6	7	42	73	21	38
miR-TG/P-P-P-P-M	111,730	33,934,931	20	15	299	291	139	118
Total	112,745	34,789,190	22	27	325	413	150	166

**Figure 5:**
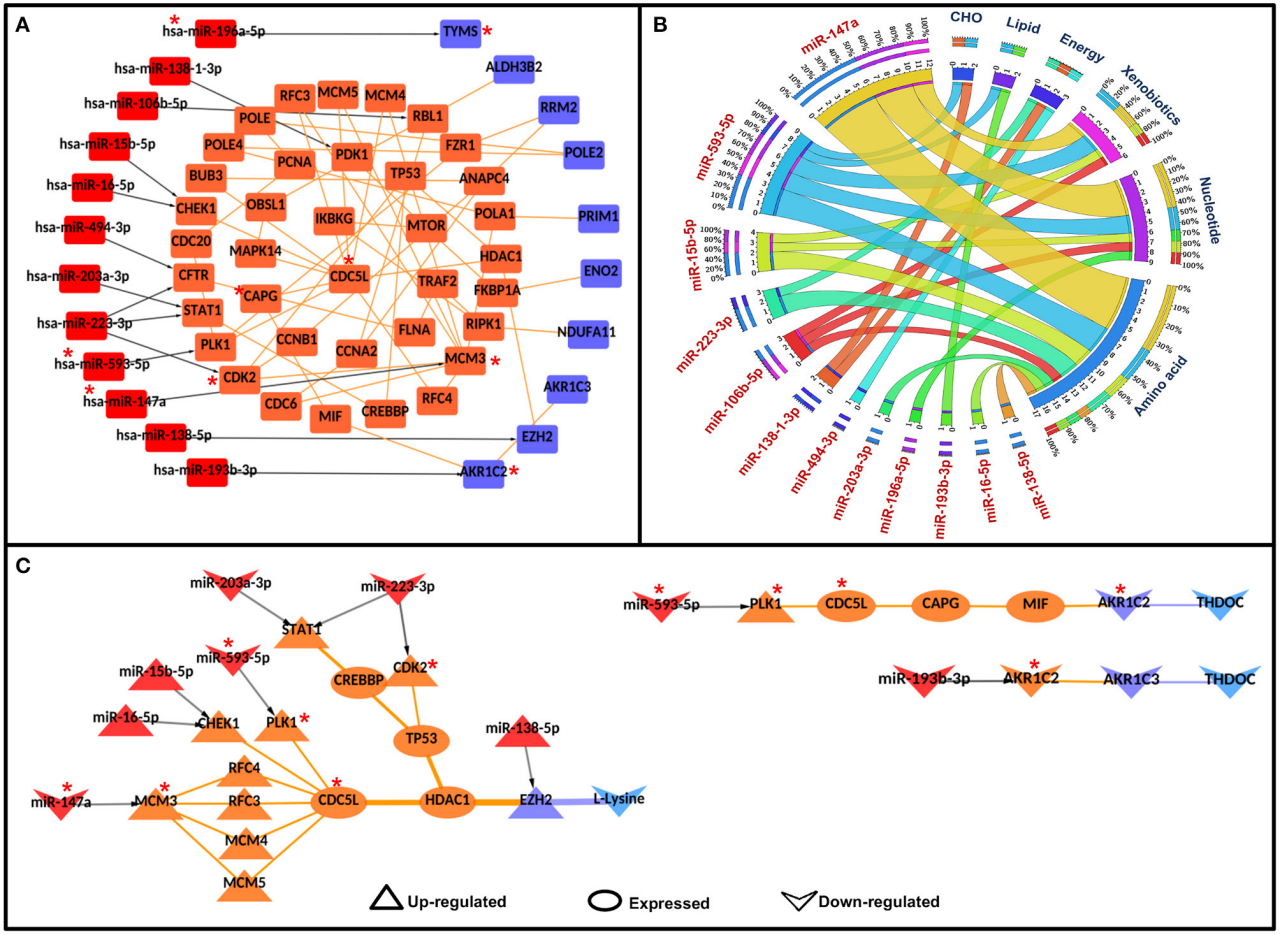
Significant miR-metabolic pathway interconnecting paths/network in cervical cancer. **(A)** represents a significant miRNA metabolic interaction network, **(B)** shows the miRNA to metabolic pathway interconnectivity, and **(C)** shows the significant miRNA to metabolic paths regulating metabolites in cervical cancer. Terminal miRNAs and metabolic enzymes are colored in red and blue. Protein-protein interactors are colored in orange. Gene regulatory edges are represented as black arrows and protein-protein interactions are represented by orange edges. Nodes with an asterisk (*) are key or effector nodes in the significant paths/network.

The nodes and edges in the CC-miRMIN were weighted based on their biological properties, differential expression status, and network topological properties in cervical cancer-specific miR-TG-PPIN. After the incorporation of HMM Model 1, 22 significant (*Z* ≥ 1) miR-M pairs were identified. HMM, Model 2, resulted in the identification of a total of 27 miR-PPI-M paths, where 22 significant miR-M pairs obtained after Model 1 were connected via their TG and PPIs. The resulting miR-PPI-M paths were converted into a network to form significant CC-miRMIN that consisted of 59 nodes and 67 interactions among them (Figure 5A).

Mapping metabolic pathway information to the terminal metabolic enzymes in significant miR-PPI-M paths showed that amino acid metabolism was highly regulated by miRNAs, followed by nucleotide metabolism, xenobiotics biodegradation and metabolism, energy metabolism, carbohydrate metabolism, and lipid metabolism (Figure 5B).

After mapping deregulated metabolites to the terminal metabolic enzymes, 13 miR-PPI-M paths were found to regulate the metabolites. L-lysine was found to be linked/regulated by miR-138-5p, miR-223-3p, miR-203a-3p, miR-593-5p, miR-15b-5p, miR-16-5p, and miR-147a via 11 miR-PPI-M paths. THDOC was found to be linked with miR-593-5p and miR-193b-3p via two different miR-PPI-M paths (Figure 5C).

### *In-silico* Perturbation of Nodes in the Final Weighted Paths/Network

*In-silico* perturbation analysis was performed to identify the paths that change significantly upon removal of a node (protein/TF/miRNA). To identify the key nodes in the final paths/networks of every module, each of the nodes present in the paths/network having *Z* ≥ 1 was removed individually from the HPPIN, and the path score was recalculated for the resulting paths/network by using HMM Models 1 and 2. Accordingly, new significant pairs and paths were identified based on Models 1 and 2, respectively. The difference of average path score (before vs. after perturbation) for each perturbed node was converted into a z-score and the nodes for which z-scores deviated from the mean as −1≥ *Z* ≥ 1 were selected as effective or key nodes in significant paths/networks. Sixteen nodes/proteins (CDC5L, PAK2, CHECK1, NDUFA9, MCM, POLA1, PIK3CA, PIK3R1, PDGFRA, LUC7L3, SERPINE1, VTN, ZRANB2, TYMS, CD14, and NDUFA11) in the signaling module (Figure 2A), 16 nodes/proteins (CDKN2A, POLA1, CDC45, CCND1, MCM5, CDC7, RRM2, MCM3, DHFR, AKR1C3, AKR1C2, AKR1C1, RRM1, TYMS, AR, and E2F1) in TF-based gene regulatory module (Figure 4A), and nine nodes/miRNA/proteins (CDK2, MCM3, mir-147a, AKR1C2, CDC5L, PLK1, mir-593-5p, TYMS, and mir-196a-5p) in miRNA-based gene regulatory module (Figure 5A) were identified as an effector or key nodes in the significant paths/networks.

### S, TF, and miR Cross-Talks in Cervical Cancer

**Figure 6:**
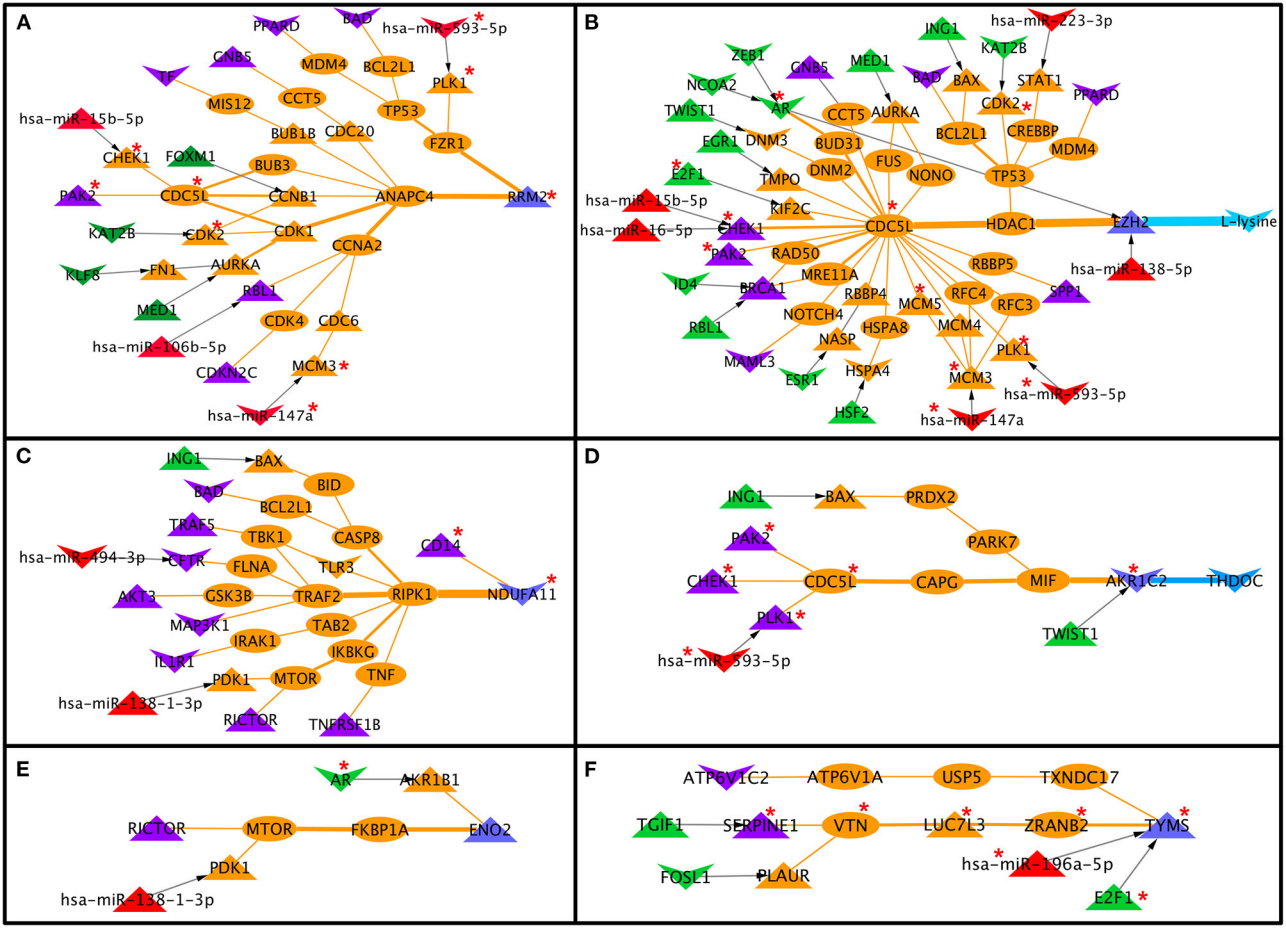
Signaling pathway proteins, transcription factor, and miR cross-connecting paths/links to metabolic enzymes in cervical cancer. Six metabolic enzymes **(A–F)** are commonly linked to signaling proteins, transcription factors, and miR. Two metabolic enzymes, EZH2 and AKR1C2 **(B,D)**, are connected to deregulated metabolites in cervical cancer. Terminal signaling pathway proteins, transcription factors, and miRNAs, metabolic enzymes are colored in purple, green, red, and blue. Protein-protein interactors are colored in orange. Gene regulatory edges are represented as black arrows and protein-protein interactions are represented by orange edges. Nodes with an asterisk (*) are key or effector nodes in the significant paths/network.

Comparing cervical cancer-specific significant S-PPI-M, TF-PPI-M, and miR-PPI-M paths or links discussed above resulted in the identification of 83 paths/links where six metabolic enzymes (RRM2, AKR1C2, ENO2, TYMS, EZH2, and NDUFA11) were probably regulated by signaling pathway proteins (BAD, PPARD, GNB5, TF, PAK2, RBL1, CDK2NC, TRAF5, CFTR, AKT3, MAP3K1, IL1R1, RICTOR, TNFRSF1B, CHEK1 BRCA1, MAML3, SPP1, PLK1, ATP6V1C2, and SERPINE1), TFs (TGIF1, FOSL1, E2F1, TWIST1, ING1, HSF2, ESR1, RBL1, ID4, EGR1, NCOA2, ZEB1, AR, MED1, KAT2B, FOXM1, and KLF8), and miRs (miR-593-5p, miR-15b-5p, miR-106b-5p, miR-147a, miR-494-3p, miR-138-1-3p, miR-196a-5p, miR-138-5p, miR-16-5p, and miR-223-3p) (Figure 6). Out of six metabolic enzymes, AKR1C2 and EZH2 were mapped to the deregulated metabolites THDOC and L-lysine, respectively (Figures 6B,D).

### Survival Analysis of the Genes/miRNAs of Identified Paths/Links in Cervical Cancer

**Figure 7:**
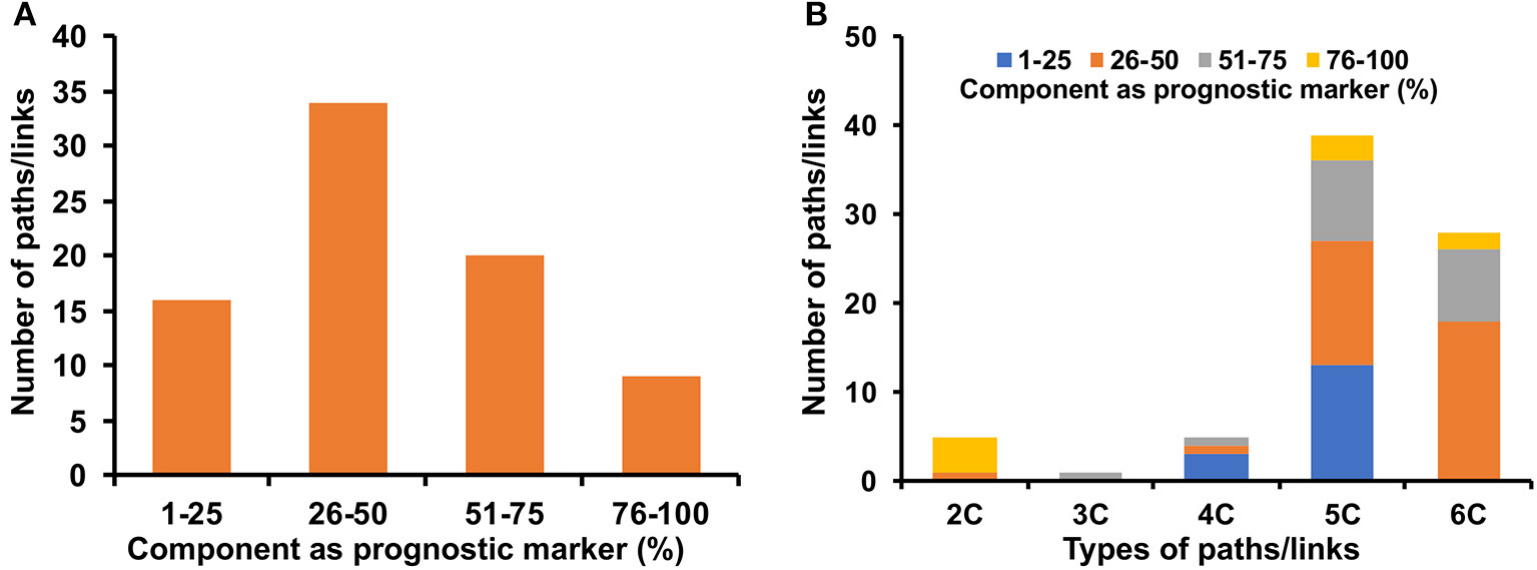
Prognostic markers in identified cross-connecting paths. **(A)** shows the fraction of components as a prognostic marker of cervical cancer in the identified paths/links. **(B)** shows the fraction of components as a prognostic marker in different types (2, 3, 4, 5, and 6 C) of identified cross-connecting paths/links.

Potential prognostic values of the genes and miRNAs of the signaling, transcriptional and post-transcriptional cross-connecting paths/links to metabolic enzymes in cervical cancer patients were explored by evaluating the correlation and OS. A total of 53 genes and 10 miRNAs were found to be significantly associated with the OS in the log-rank test with a *p* < 0.05 (Supplementary Table 12). Mapping these genes and miRNAs onto the corresponding cross-connecting paths/links yielded 16, 34, 20, and 9 paths/links to have 1–25, 26–50, 51–75, and 76–100% of their component as a prognostic marker in cervical cancer patients (Figure 7A). Almost all the final selected paths/links (79 out of 83) possess at least one node (gene/miRNA), whose expression is significantly associated with cervical cancer patients' survival. In total, 38% (30 out of 79) of the selected paths/links have more than 50% nodes to be significant (*p* < 0.05) prognosis marker (Figure 7A). Further, we checked the status of these prognostic markers in different types of paths/links. Most of the two-component (2C) paths were found to have 100% of their component as a prognostic marker. Three-component (3C) paths were found to have 51–75% of their component nodes as a prognostic marker. Similarly, significantly higher numbers of longer or higher component paths (e.g., 4, 5, and 6C, respectively) also possess more than 25% of their nodes as a prognostic marker (Figure 7B).

### S, TF, and miR Cross-Talks in Breast and Ovarian Cancers

To investigate whether the cross-pathway links are specific to cervical cancer, we identified such paths from two other female-specific cancers, such as breast and ovarian cancers. As mentioned in the Methods, paths originating from S, TF, and miR connecting metabolic enzymes (M) were identified in breast and ovarian cancers using the cancer-specific transcriptomics data mapping followed by implementation of HMM-based mathematical models. A total of 2,79, 311 and 413 S-PPI-M, TF-PPI-M, and miR-PPI-M paths were identified in breast cancer connecting 232, 261 and 325 S-M, TF-M, and miR-M pairs, respectively. Similarly, 250, 669 and 166 S-PPI-M, TF-PPI-M, and miR-PPI-M paths were identified in ovarian cancer connecting 218, 577 and 150 S-M, TF-M, and miR-M pairs, respectively (Tables 1–3). Mapping of deregulated metabolites resulted in 69 paths in breast cancer and 481 paths in ovarian cancer.

**Figure 8:**
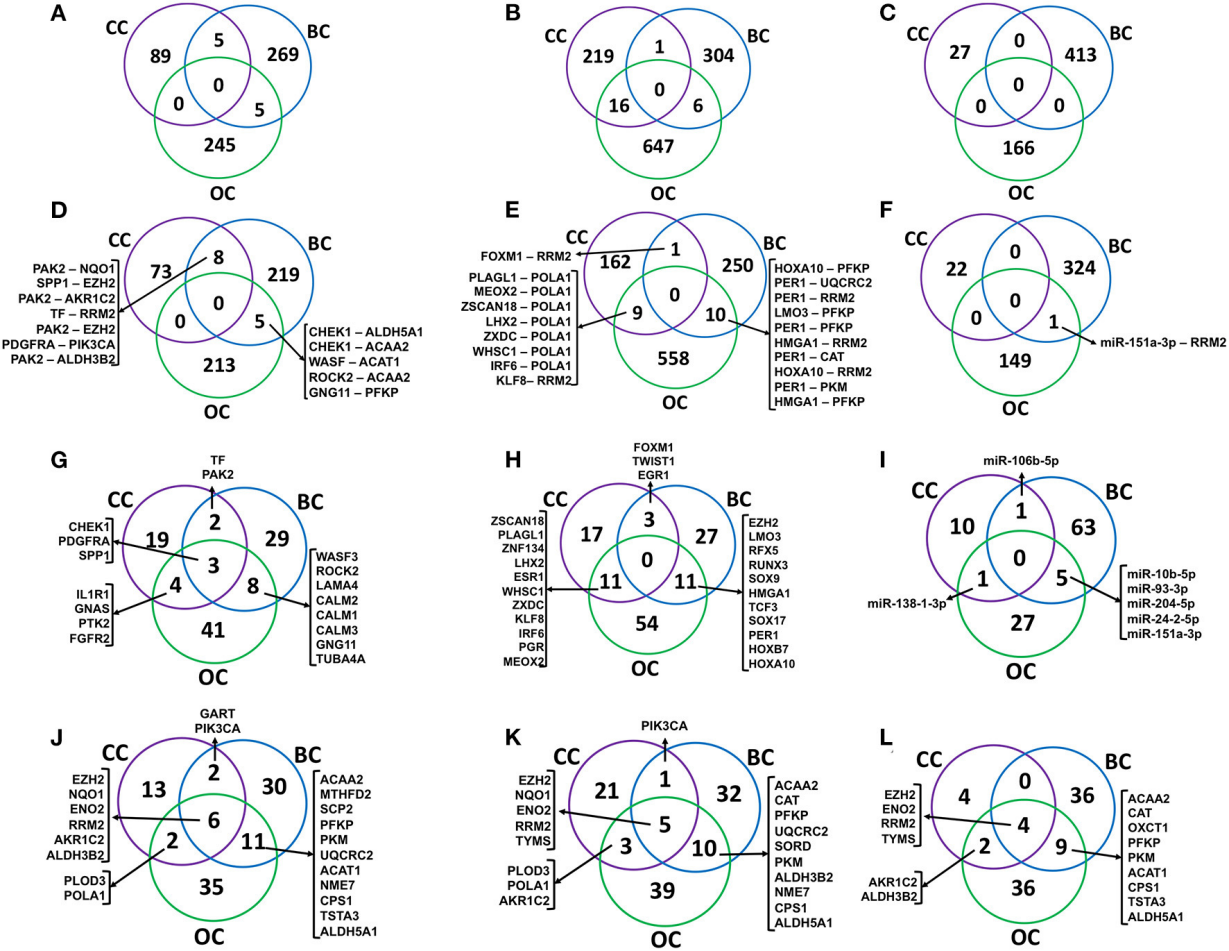
Comparison of regulatory molecules and links in female-specific cancer. **(A–C)** show the overlap of significant signaling proteins (S), transcription factor (TF), and miR to metabolic enzyme connecting paths, respectively, identified from cervical cancer (CC), breast cancer (BC), and ovarian cancer (OC) specific networks. **(D–F)** show the overlap of S-M, TF-M, and miR-M pairs, respectively. **(G–I)** represent the overlap of terminal signaling protein in S–M paths, a transcription factor in TF–M paths, and miR–M paths, respectively. **(J–L)** represent an overlap of terminal metabolic enzymes. S-M, signaling-metabolic; TF-M, TF-metabolic; miR-M, microRNA-metabolic.

The signaling (S-M), transcriptional (TF-M), and post-transcriptional (miR-M) regulatory links identified from cervical, breast, and ovarian cancer networks were compared to estimate the common and specific regulators and regulatory links (Figure 8). Interestingly, a very little overlap of regulatory paths and pairs was observed among the three types of cancers (Figures 8A–F). In total, 32% of terminal signaling proteins and 43% of terminal metabolic enzymes forming CC-specific S-M enzymes paths were found to be common with that extracted from breast and ovarian cancer networks (Figures 8G,J). Similarly, 46% of terminal TFs and 30% of terminal metabolic enzymes forming CC-specific TF-metabolic enzyme paths were found to be common with those extracted from breast and ovarian cancer networks (Figures 8H,K). Three metabolic enzymes (EZH2, ENO2, and RRM2) were found to be commonly regulated by S-M, TF-M, and miR in all three cancers (Figure 8).

The metabolic enzymes EZH2 and MIF connected to deregulated metabolites L-lysine and citric acid were commonly regulated in breast cancer. However, 17 metabolic enzymes (EZH2, MTHFD1, ALDH3B2, ATP6V1B1, TCIRG1, AGMAT, SETDB1, PFKL, TKT, INPPL1, MTHFD2, CPS1, MARS, PFKP, AASDHPPT, ATP6V0D2, and PLOD3) connected to nine deregulated metabolites (L-lysine, adenosine monophosphate, fructose 6-phosphate, homovanillic acid, methylimidazole acetic acid, N-acetylglutamic acid, phenylacetic acid, phosphate, and Urea) were found to be commonly regulated in ovarian cancer.

The metabolic enzyme EZH2 was connected to deregulated metabolites L-lysine in cervical, breast, and ovarian cancer (Figure 6B, Supplementary Figure 6). Twenty-five out of the 27 paths/links connected to metabolic enzyme EZH2 in the breast cancer network (Supplementary Figure 6A) possesses at least one gene, whose expression is significantly associated with drug/chemotherapy response. Seventeen out of the 37 genes associated with these paths were predicted as cancer biomarkers with potential clinical utility (AUC ≥ 0.6; Supplementary Table 13).

## Discussion

Understanding the molecular mechanisms for cancer progression and subsequent development of potential therapeutics to inhibit this complex disease are difficult from the independent knowledge of ongoing signaling, gene regulatory, and metabolic alterations. Therefore, understanding the intricate coordination of signaling and gene regulatory-induced proliferation of tumor cells/growth and metabolic processes is very much required. An integrated view of the probable interconnections between oncogenic signaling-gene regulatory pathways and the metabolic shift could be one of the better ways to find out possible potential therapeutic targets. Our approach toward the establishment of a cross-pathway metabolic interconnection network is an attempt in that direction.

It is well-established that genetic modifications, altered transcriptional, and post-transcriptional regulations are responsible for mediating the changes in biological processes, which ultimately shape complex pathophysiological situations like cancer. The interconnectivity and regulations are perhaps maintained through the systemic-coordinated interaction of proteins as a complex system, acting as a perfect molecular machine (62–64). Therefore, the identification of such a precise protein-interaction network responsible for the disease progression is of utmost importance for understanding the disease and potential therapeutic development.

In this study, we have developed a biology framework of a cervical cancer-specific system where signaling (S) pathway proteins, miRNA, and TF-based gene-regulatory modules are connected to metabolic (M) pathway proteins through PPIs. Publicly available transcriptomic data derived from cervical cancer patients were incorporated into a mathematical modeling set-up to weigh and rank the interconnecting link/paths in addition to biological and network topological properties to extract out high confidence inter-pathway connections that are perhaps responsible for facilitating metabolic adaptation in cervical cancer.

In our previous study, we implemented the underlined mathematical model-based approach for the development, test, and validation of S-M interconnecting links using glioblastoma multiform (GBM) cell line-derived transcriptomics and proteomics data (37). Further, *in-vitro* perturbation of genes/proteins involved in forming a high-score interconnection between S-M pathway proteins showed a significant change in the expression of proteins involved in the metabolic pathway. This validated our model for discovering hitherto unknown connections/involvement between signaling and metabolic genes/proteins. As a natural follow-up study, here we have significantly upgraded the previous model with two entirely new types of connectivity paths linking TF and miRNA-based regulatory mechanisms to altered states of metabolic enzymes. We have used large-scale patient-derived cervical cancer data and implemented additional network topology-based weights to signify the identified cross-pathway links. Further, we have utilized differential metabolite data to extract out paths that correlate with the altered status of the metabolic enzymes that were proposed to be regulated via signaling and regulatory factors. Comparison of the significant paths originated with S, TFs, and miRNAs yielded 88 commonly linked paths connecting six common metabolic enzymes (e.g., RRM2, AKR1C2, ENO2, TYMS, EZH2, and NDUFA11).

The ribonucleotide reductase subunit M2 (RRM2) was found to be significantly upregulated in cervical cancer tissue and is linked to promoting the progression of cervical cancer (65). RRM2 is likely to become a novel potential diagnostic and prognostic biomarker of cervical cancer.

Aldo-keto reductase subfamily 1C2, which plays a major role in regulating the activity of androgens, estrogens, progesterone, and prostaglandins metabolisms, is also implicated with cervical, endometrial, and bladder cancers (66, 67). Overexpression of AKR1C2 is found to be a mildly favorable prognostic marker (Supplementary Table 12) but lower expression of NADH:ubiquinone oxidoreductase subunit A11 (NDUFA11) is prognostically unfavorable in cervical cancer (Supplementary Table 12). Enolase 2 (ENO2) and thymidylate synthetase (TYMS) are found to be upregulated in cervical cancer transcription datasets (68, 69). Overexpression of enhancer zeste homolog 2 (EZH2) has been linked with proliferation, progression, and prognosis of cervical cancer (70). However, our survival analysis using data of cervical cancer patients from TCGA suggested much lower survival with lower expression of EZH2 (Supplementary Table 12).

Several miRNAs have been identified whose roles have been implicated in cervical cancer progression. Most of the miRNAs except one (miR-593-5p) forming the metabolic pathway PPI links were previously reported to be dysregulated in cervical cancers (71–75). However, the diverse mechanisms by which these miRNAs could regulate cervical cancer progression were not well-known especially their roles in regulating the metabolic adaptation in cervical cancer cells. Our study provides novel avenues to study the impact of these important miRNAs in the regulation of metabolic reprogramming in cervical cancer. miR-593 plays important role in the regulation of lung, breast, and gastric cancer proliferation (76–79). Higher expression of miR-593 is found to be unfavorable for the survival of cervical cancer patients (Supplementary Table 12). Hence, its role in cervical cancer especially in its metabolic adaptation is worth investigating further.

Transcription factors are key regulators of cancer proliferation and metastasis. Roles of several transcription factors, such as SOX2, E2F4, E2F1, POU5F1, SMAD3, SMAD2, VDR, ERG, TP53, EWS, c-fos, fra-1, OCT4, KLF4, C-MYC, and NANOG, were established in cervical cancer (80, 81). Our study highlights the probable roles of important transcription factors in regulating the metabolic status of cervical cancer cells via modulating the metabolic enzymes. Thirty-one TFs were found to be connected to 30 metabolic enzymes via the TG and their respective PPIs. Fifteen TFs were found to be linked with six metabolic enzymes for which altered metabolite status could be associated (Figure 4).

Ras, cell cycle, MAPK, EGFR, and p53 are among the top five most connected signaling pathways to the metabolic enzymes via PPI interconnectivity (Figure 3). Among the 27 terminal signaling proteins that form significant connections with metabolic enzymes, 9 (CHEK1, MAP3K1, CDKN2C, PAK2, EGFR, FGFR2, PDGFRA, and PTK2) are found to be kinases. TFs and miRNAs are generally regarded as “undruggable,” hence, these regulatory kinases could be ideal candidates for targets of small molecules inhibitors/drugs to check their roles in altering the functional activities of the connected enzymes.

All cervical cancer-based cross-pathway links are provided in Supplementary Tables 8–11. Similarly, an online platform is also created as a separately published work (82) where cervical cancer dataset-specific S-M, TF-metabolic, miRNA-metabolic, and combined paths are made available at http://www.hpppi.iicb.res.in/APODHIN/home.html.

Cervical cancer-based PPI links to metabolic enzymes originated from signaling (S), TF, and miR regulatory molecules were also compared to the same identified from breast and ovarian cancer networks. Comparison of the regulators and regulatory links yielded little overlap among the three cancers (Figure 8) indicating the existence of cancer-specific regulatory mechanisms for probable metabolic alterations. However, some signaling proteins were found to be common regulatory molecules among the three cancers whereas terminal enzymes, such as EZH2, ENO2, RRM2, and TYMS, were found to be commonly regulated in all the cancers by three different regulatory mechanisms (Figure 8).

We understand that our approach is computation heavy and our findings require further *in vitro* and *in vivo* experimental validations. Similarly, for effective stratification of the patients, multiple omics data (e.g., transcriptomics, proteomics, and metabolomics) need to be generated from an individual patient. Nevertheless, we believe that our systems biology-based approach of identifying multi-factor signature links connected to the regulation of status and functionalities of metabolic enzymes paves the way for future studies which could be aimed toward identifying novel regulators of metabolic alterations in cervical cancer.

## Data Availability Statement

Publicly available datasets were analyzed in this study. This data can be found at: Gene expression omnibus (GEO).

## Author Contributions

SC conceptualized the project. KK and SB performed the analysis. KK and SC analyzed the data and drafted the manuscript. All authors contributed to the article and approved the submitted version.

## Funding

SC acknowledges the Systems Medicine Cluster (SyMeC) Grant (GAP357), Department of Biotechnology (DBT) for funding. KK acknowledges the Department of Biotechnology (DBT) for fellowship (DBT/2016/IICB/491). SB acknowledges the Council of Scientific and Industrial Research (CSIR) for fellowship grant [31/002(1090)/2017-EMR-I].

## Conflict of Interest

The authors declare that the research was conducted in the absence of any commercial or financial relationships that could be construed as a potential conflict of interest.

## Publisher's Note

All claims expressed in this article are solely those of the authors and do not necessarily represent those of their affiliated organizations, or those of the publisher, the editors and the reviewers. Any product that may be evaluated in this article, or claim that may be made by its manufacturer, is not guaranteed or endorsed by the publisher.

## References

[1] Bray F Ferlay J Soerjomataram I Siegel RL Torre LA Jemal A. Global cancer statistics 2018: GLOBOCAN estimates of incidence and mortality worldwide for 36 cancers in 185 countries. CA Cancer J Clin. (2020) 68:394–424. 10.3322/caac.21492 30207593

[2] World Health O.rganization. Human Papillomavirus (HPV) and Cervical Cancer. (2020). Available online at: https://www.who.int/en/news-room/fact-sheets/detail/human-papillomavirus-(hpv)-and-cervical-cancer (accessed June 10, 2020).

[3] Anttila T Saikku P Koskela P Bloigu A Dillner J Ikäheimo I. Serotypes of *Chlamydia trachomatis* and risk for development of cervical squamous cell carcinoma. J Am Med Assoc. (2001) 285:47–51. 10.1001/jama.285.1.47 11150108

[4] Jee B Yadav R Pankaj S Shahi SK. Immunology of HPV-mediated cervical cancer: current understanding. Int Rev Immunol. (2020) 40:359–78. 10.1080/08830185.2020.1811859 32853049

[5] Walboomers JM Jacobs MV Manos MM Bosch FX Kummer JA Shah KV. Human papillomavirus is a necessary cause of invasive cervical cancer worldwide. J Pathol. (1999) 189:12–9. 10.1002/(SICI)1096-9896(199909)189:1<12::AID-PATH431>3.0.CO;2-F 10451482

[6] Munoz N Bosch FX de Sanjose S Herrero R Castellsagué X. Shah KV. Epidemiologic classification of human papillomavirus types associated with cervical cancer. N Engl J Med. (2003) 348:518–27. 10.1056/NEJMoa021641 12571259

[7] de Sanjose S Quint WG Alemany L Geraets DT Klaustermeier JE Lloveras B. Human papillomavirus genotype attribution in invasive cervical cancer: a retrospective cross-sectional worldwide study. Lancet Oncol. (2010) 11:1048–56. 10.1016/S1470-2045(10)70230-8 20952254

[8] Haedicke J Iftner T. Human papillomaviruses and cancer. RadiotherOncol. (2013) 108:397–402. 10.1016/j.radonc.2013.06.004 23830197

[9] Groves IJ Coleman N. Pathogenesis of human papillomavirus-associated mucosal disease. J Pathol. (2015) 235:527–38. 10.1002/path.4496 25604863

[10] Narisawasaito M Kiyono T. Basic mechanisms of high-risk human papillomavirus-induced carcinogenesis: roles of E6 and E7 proteins. Cancer Sci. (2007) 98:1505–11. 10.1111/j.1349-7006.2007.00546.x 17645777 PMC11158331

[11] Hanahan D Weinberg RA. Hallmarks of cancer: the next generation. Cell. (2011) 144:646–74. 10.1016/j.cell.2011.02.013 21376230

[12] Hanahan D Weinberg RA. The hallmarks of cancer. Cell. (2000) 100:57–70. 10.1016/S0092-8674(00)81683-9 10647931

[13] Ward PS Thompson CB. Signaling in control of cell growth and metabolism. Cold Spring Harbor Perspect Biol. (2012) 4:7a006783. 10.1101/cshperspect.a006783 22687276 PMC3385956

[14] Mo Y Wang Y Zhang L Yang L Zhou M Li X. The role of Wnt signaling pathway in tumor metabolic reprogramming. J Cancer. (2019) 10:3789–97. 10.7150/jca.31166 31333796 PMC6636296

[15] Papa S Choy PM Bubici C. The ERK and JNK pathways in the regulation of metabolic reprogramming. Oncogene. (2019) 38:2223–40. 10.1038/s41388-018-0582-8 30487597 PMC6398583

[16] Martin-Martin N Carracedo A Torrano V. Metabolism and transcription in cancer: merging two classic tales. Front Cell Dev Biol. (2017) 5:119. 10.3389/fcell.2017.00119 29354634 PMC5760552

[17] Dong Y Tu R Liu H Quing G. Regulation of cancer cell metabolism: oncogenic MYC in the driver's seat. Sig Transduct Target Ther. (2020) 5:124. 10.1038/s41392-020-00235-2 32651356 PMC7351732

[18] Machida K. Pluripotency transcription factors and metabolic reprogramming of mitochondria in tumor-initiating stem-like cells. Antioxid Redox Signal. (2018) 28:1080–9. 10.1089/ars.2017.7241 29256636 PMC5865250

[19] Rottiers V Naar AM. MicroRNAs in metabolism and metabolic disorders. Nat Rev Mol Cell Biol. (2012) 13:239–50. 10.1038/nrm3313 22436747 PMC4021399

[20] Chen B Li H Zeng X Yang P Liu X Zhou X. Roles of microRNA on cancer cell metabolism. J Transl Med. (2012) 10:228. 10.1186/1479-5876-10-228 23164426 PMC3563491

[21] Singh PK Mehla K Hollingsworth MA Johnson KR. Regulation of aerobic glycolysis by microRNAs in cancer. Mol Cell Pharmacol. (2011) 3:125–34. 22792411 PMC3392682

[22] Ji Z Su J Liu C Wang H Huang D Zhou X. Integrating genomics and proteomics data to predict drug effects using binary linear programming. PLoS ONE. (2014) 9:e102798. 10.1371/journal.pone.0102798 25036040 PMC4103865

[23] Cheng F Murray JL Zhao J Sheng J Zhou Z Rubin DH. Systems biology-based investigation of cellular antiviral drug targets identified by gene-trap insertional mutagenesis. PLOS Comput Biol. (2016) 12:e1005074. 10.1371/journal.pcbi.1005074 27632082 PMC5025164

[24] Puniya BL Kulshreshtha D Mittal I Mobeen A Ramachandran S. Integration of metabolic modeling with gene co-expression reveals transcriptionally programmed reactions explaining robustness in mycobacterium tuberculosis. Sci Rep. (2016) 6:23440. 10.1038/srep24916 27000948 PMC4802306

[25] Puniya BL Allen L Hochfelder C Majumder M Helikar T. Systems perturbation analysis of a large-scale signal transduction model reveals potentially influential candidates for cancer therapeutics. Front Bioeng Biotechnol. (2016) 4:10. 10.3389/fbioe.2016.00010 26904540 PMC4750464

[26] Samaga R Klamt S. Modeling approaches for qualitative and semi-quantitative analysis of cellular signaling networks. Cell Commun Signal. (2013) 11:43. 10.1186/1478-811X-11-43 23803171 PMC3698152

[27] Albert R Thakar J. Boolean modeling: a logic-based dynamic approach for understanding signaling and regulatory networks and for making useful predictions. Wiley Interdiscip Rev SystBiol Med. (2014) 6:353–69. 10.1002/wsbm.1273 25269159

[28] Le Novère N. Quantitative and logic modelling of molecular and gene networks. Nat Rev Genet. (2015) 16:146–58. 10.1038/nrg3885 25645874 PMC4604653

[29] Naldi A Monteiro PT Müssel C Consortium for Logical M.odels, Tools.. Cooperative development of logical modelling standards and tools with CoLoMoTo. Bioinformatics. (2015) 31:1154–9. 10.1093/bioinformatics/btv013 25619997

[30] Saez-Rodriguez J Alexopoulos LG Zhang M Morris MK Lauffenburger DA Sorger PK. Comparing signaling networks between normal and transformed hepatocytes using discrete logical models. Cancer Res. (2011) 71:5400–11. 10.1158/0008-5472.CAN-10-4453 21742771 PMC3207250

[31] Schoeberl B Eichler-Jonsson C Gilles ED Müller G. Computational modeling of the dynamics of the MAP kinase cascade activated by surface and internalized EGF receptors. Nat Biotechnol. (2002) 20:370–5. 10.1038/nbt0402-370 11923843

[32] Huang Z Mayr NA Yuh WT Lo SS Montebello JF Grecula JC. Predicting outcomes in cervical cancer: a kinetic model of tumor regression during radiation therapy. Cancer Res. (2010) 70:463–70. 10.1158/0008-5472.CAN-09-2501 20068180 PMC2822442

[33] Chaouiya C. Petri net modelling of biological networks. Brief Bioinform. (2007) 8:210–9. 10.1093/bib/bbm029 17626066

[34] Hautaniemi S Kharait S Iwabu A Wells A Lauffenburger DA. Modeling of signal-response cascades using decision tree analysis. Bioinformatics. (2005) 21:2027–35. 10.1093/bioinformatics/bti278 15657095

[35] Peng H Zhao W Tan H Ji Z Li J Li K. Prediction of treatment efficacy for prostate cancer using a mathematical model. Sci Rep. (2016) 6:21599. 10.1038/srep21599 26868634 PMC4751505

[36] Orth JD Thiele I Palsson BO. What is flux balance analysis? Nat Biotechnol. (2010) 28:245–8. 10.1038/nbt.1614 20212490 PMC3108565

[37] Bag AK Mandloi S Jarmalavicius S Mondal S Kumar K Mandal C. Connecting signaling and metabolic pathways in EGF receptor-mediated oncogenesis of glioblastoma. PLoS Comput Biol. (2019) 15:e1007090. 10.1371/journal.pcbi.1007090 31386654 PMC6684045

[38] Bhattacharyya M Chakrabarti S. Identification of important interacting proteins (IIPs) in *Plasmodium falciparum* using large-scale interaction network analysis and in-silico knock-out studies. Malar J. (2015) 14:70. 10.1186/s12936-015-0562-1 25879642 PMC4333160

[39] Edgar R Domrachey M Lash AE. Gene expression omnibus: NCBI gene expression and hybridization array data repository. Nucleic Acids Res. (2002) 30:207–10. 10.1093/nar/30.1.207 11752295 PMC99122

[40] Smyth GK. Linear models and empirical bayes methods for assessing differential expression in microarray experiments. Stat Applic Genet Mol Biol. (2004) 3:3. 10.2202/1544-6115.1027 16646809

[41] Smyth GK. Limma: linear models for microarray data. In: eds Gentleman R Carey V Dudoit S Irizarry R Huber W editors. Bioinformatics and Computational Biology Solutions Using R and Bioconductor. New York, NY: Springer (2005). p. 397–420. 10.1007/0-387-29362-0_23

[42] Davis S Meltzer PS. GEOquery: a bridge between the gene expression omnibus (GEO) and bioconductor. Bioinformatics. (2007) 23:1846–7. 10.1093/bioinformatics/btm254 17496320

[43] Benjamini Y Hochberg Y. Controlling the false discovery rate: a practical and powerful approach to multiple testing. J R Stat Soc Ser B. (1995) 57:289–300. 10.1111/j.2517-6161.1995.tb02031.x

[44] Szklarczyk D Gable AL Lyon D Junge A Wyder S Huerta-Cepas J. STRING v11: protein-protein association networks with increased coverage, supporting functional discovery in genome-wide experimental datasets. Nucleic Acids Res. (2019) 47:D607–13. 10.1093/nar/gky1131 30476243 PMC6323986

[45] Bovolenta LA Acencio ML Lemke N. HTRIdb: an open-access database for experimentally verified human transcriptional regulation interactions. BMC Genomics. (2012) 13:405. 10.1186/1471-2164-13-405 22900683 PMC3472291

[46] Han H Cho JW Lee S Yun A Kim H Bae D. TRRUST v2: an expanded reference database of human and mouse transcriptional regulatory interactions. Nucleic Acids Res. (2018) 46:D380–6. 10.1093/nar/gkx1013 29087512 PMC5753191

[47] Huang HY Lin YC Li J Huang KY Shrestha S Hong HC. miRTarBase 2020: updates to the experimentally validated microRNA–target interaction database. Nucleic Acids Res. (2020) 48:D148–4. 10.1093/nar/gkz896 31647101 PMC7145596

[48] Karagkouni D Paraskevopoulou MD Chatzopoulos S Vlachos IS Tastsoglou S. DIANA-TarBase v8:a decade-long collection of experimentally supported miRNA-gene interaction. Nucleic Acids Res. (2018) 46:D239–45. 10.1093/nar/gkx1141 29156006 PMC5753203

[49] Hagberg AA Schult DA Swart PJ. Exploring network structure, dynamics, and function using NetworkX. In: Varoquaux G Vaught T Millman J editors. Proceedings of the 7th Python in Science Conference (SciPy2008). Pasadena, CA (2008). p. 11–15.

[50] Liao Y Wang J Jaehnig E Shi Z Zhang B. WebGestalt 2019: gene set analysis toolkit with revamped UIs and APIs. Nucleic Acids Res. (2019) 47:W199–205. 10.1093/nar/gkz401 31114916 PMC6602449

[51] Kanehisa M Furumichi M Tanabe M Sato Y Morishima K. KEGG: new perspectives on genomes, pathways, diseases and drugs. Nucleic Acids Res. (2017) 45:D353–61. 10.1093/nar/gkw1092 27899662 PMC5210567

[52] Kanehisa M Goto S. KEGG: kyoto encyclopedia of genes and genomes. Nucleic Acids Res. (2000) 28:27–30. 10.1093/nar/28.1.27 10592173 PMC102409

[53] Shannon P Markiel A Ozier O Baliga NS Wang JT Ramage D. Cytoscape:a software environment for integrated models of biomolecular interaction networks. Genome Res. (2003) 13:2498–504. 10.1101/gr.1239303 14597658 PMC403769

[54] Krzywinski M Schein J Birol I Connors J Gascoyne R Horsman D. Circos: an information aesthetic for comparative genomics. Genome Res. (2009) 19:1639–45. 10.1101/gr.092759.109 19541911 PMC2752132

[55] Yang K Xia B Wang W Cheng J Yin M Xie H. A comprehensive analysis of metabolomics and transcriptomics in cervical cancer. Sci Rep. (2017) 7:43353. 10.1038/srep43353 28225065 PMC5320559

[56] Park J Shin Y Kim TH Kim DH Lee A. Plasma metabolites as possible biomarkers for diagnosis of breast cancer. PLoS ONE. (2019) 14:e0225129. 10.1371/journal.pone.0225129 31794572 PMC6890236

[57] Turkoglu O Zeb A Graham S Szyperski T Szender JB Odunsi K. Metabolomics of biomarker discovery in ovarian cancer: a systematic review of the current literature. Metabolomics. (2016) 12:60. 10.1007/s11306-016-0990-0 28819352 PMC5557039

[58] Wishart DS Feunang YD Marcu A Guo AC Liang K Vazquez-Freshno R. HMDB 4.0 – the human metabolome database for (2018). Nucleic Acids Res. (2018) 46:D608–17. 10.1093/nar/gkx1089 29140435 PMC5753273

[59] Gyorffy B. Survival analysis across the entire transcriptome identifies biomarkers with the highest prognostic power in breast cancer. Comput Struc Biotechnol J. (2021) 19:4101–9. 10.1016/j.csbj.2021.07.014 34527184 PMC8339292

[60] Nagy A Munkacsy G Gyorffy B. Pancancer survival analysis of cancer hallmark genes. Sci Rep. (2021) 11:6047. 10.1038/s41598-021-84787-5 33723286 PMC7961001

[61] Fekete J Gyorffy B. ROCplot.org: Validating predictive biomarkers of chemotherapy/hormonal therapy/anti-Her2 therapy using transcriptomic data of 3,104 breast cancer patients. Int J Cancer. (2019) 145:3140–51. 10.1002/ijc.32369 31020993

[62] Vidal M Cusick ME Barabási AL. Interactome networks and human disease. Cell. (2011) 144:986–98. 10.1016/j.cell.2011.02.016 21414488 PMC3102045

[63] Sevimoglu T Arga KY. The role of protein interaction networks in systems biomedicine. Comput Struct Biotechnol J. (2014) 11:22–7. 10.1016/j.csbj.2014.08.008 25379140 PMC4212283

[64] Wang J Yi Y Chen Y Xiong Y Zhang W. Potential mechanism of RRM2 for promoting cervical cancer based on weighted gene co-expression network analysis. Int J Med Sci. (2020) 17:2362–72. 10.7150/ijms.47356 32922202 PMC7484645

[65] RiŽner TL. Enzymes of the AKR1B and AKR1C subfamilies and uterine diseases. Front Pharmacol. (2012) 3:34. 10.3389/fphar.2012.00034 22419909 PMC3301985

[66] Tai HL Lin TS Huang HH Lin TY Chou MC Chiou SH. Overexpression of aldo-keto reductase 1C2 as a high-risk factor in bladder cancer. Oncol Rep. (2007) 17:305–11. 10.3892/or.17.2.305 17203165

[67] Liu D Mao Y Chen C Zhu F Lu W Ma H. Expression patterns and clinical significances of ENO2 in lung cancer: an analysis based on oncomine database. Ann Transl Med. (2020) 8:639. 10.21037/atm-20-3354 32566576 PMC7290642

[68] Yang HJ Xue JM Li J Wan LH Zhu YX. Identification of key genes and pathways of diagnosis and prognosis in cervical cancer by bioinformatics analysis. Mol Genet Genom Med. (2020) 8:e1200. 10.1002/mgg3.1200 32181600 PMC7284022

[69] Liu Y Liu T Bao X He M Li L Yang X. Increased EZH2 expression is associated with proliferation and progression of cervical cancer and indicates a poor prognosis. Int J Gynecol Pathol. (2014) 33:218–24. 10.1097/PGP.0b013e31829c6574 24681730

[70] Sharma G Dua P Agarwal SM. A comprehensive review of dysregulated miRNAs involved in cervical cancer. Curr Genomics. (2014) 15:310–23. 10.2174/1389202915666140528003249 25132800 PMC4133953

[71] Wang JY Chen LJ. The role of miRNAs in the invasion and metastasis of cervical cancer. Biosci Rep. (2019) 39:BSR20181377. 10.1042/BSR20181377 30833362 PMC6418402

[72] Pardini B De Maria D Francavilla A Di Gaetano C Ronco G Naccarati A. MicroRNAs as markers of progression in cervical cancer: a systematic review. BMC Cancer. (2018) 18:696. 10.1186/s12885-018-4590-4 29945565 PMC6020348

[73] Ma Z Cai Y Zhang L Tian C Lyu L. LINC00319 promotes cervical cancer progression via targeting miR-147a/IGF1R pathway. Cancer Biother Radiopharm. (2020). 10.1089/cbr.2020.3722. [Epub ahead of print]. 32644822

[74] Wang F Zhang H Xu N Huang N Tian C Ye A. A novel hypoxia-induced miR-147a regulates cell proliferation through a positive feedback loop of stabilizing HIF-1α. Cancer Biol Ther. (2016) 17:790–8. 10.1080/15384047.2016.1195040 27260617 PMC5004686

[75] Wei F Wang M Li Z Wang Y Zhou Y. miR-593 inhibits proliferation and invasion and promotes apoptosis in non-small cell lung cancer cells by targeting SLUG-associated signaling pathways. Mol Med Rep. (2019) 20:5172–82. 10.3892/mmr.2019.10776 31661137 PMC6854539

[76] Han W Wang L Zhang L Wang Y Li Y. Circular RNA circ-RAD23B promotes cell growth and invasion by miR-593-3p/CCND2 and miR-653-5p/TIAM1 pathways in non-small cell lung cancer. Biochem Biophys Res Commun. (2019) 510:462–6. 10.1016/j.bbrc.2019.01.131 30722989

[77] Song L Xiao Y. Downregulation of hsa_circ_0007534 suppresses breast cancer cell proliferation and invasion by targeting miR-593/MUC19 signal pathway. Biochem Biophys Res Commun. (2018) 503:2603–10. 10.1016/j.bbrc.2018.08.007 30139516

[78] Dong L Hong H Chen X Huang Z Wu W Wu F. LINC02163 regulates growth and epithelial-to-mesenchymal transition phenotype via miR-593-3p/FOXK1 axis in gastric cancer cells. Artif Cells Nanomed Biotechnol. (2018) 46:607–15. 10.1080/21691401.2018.1464462 29893595

[79] Yu H Wei W Cao W Zhan Z Yan L Wu K. Regulation of cell proliferation and metastasis by microRNA-593-5p in human gastric cancer. Onco Targets Ther. (2018) 11:7429–40. 10.2147/OTT.S178151 30425531 PMC6204852

[80] Sinkala M Zulu M Nkhoma P Kafita D Zulu E Tembo R. A systems approach identifies key regulators of hpv-positive cervical cancer. medRxiv [Preprint]. (2020). 10.1101/2020.05.12.20099424

[81] Ruiz G Valencia-González HA Pérez-Montiel D Muñoz F Ocadiz-Delgado R Fernández-Retana J. Genes involved in the transcriptional regulation of pluripotency are expressed in malignant tumors of the uterine cervix and can induce tumorigenic capacity in a nontumorigenic cell line. Stem Cells Int. (2019) 2019:7683817. 10.1155/2019/7683817 31885625 PMC6914900

[82] Biswas N Kumar K Bose S Bera R Chakrabarti S. Analysis of pan-omics data in human interactome network (APODHIN). Front Genet. (2020) 11:589231. 10.3389/fgene.2020.589231 33363571 PMC7753071

