# Supplementary material for: Identification of Cross-Pathway Connections via Protein-Protein Interactions Linked to Altered States of Metabolic Enzymes in Cervical Cancer
Source: Front Med (Lausanne). 2021 Nov 1;8:736495. doi: 10.3389/fmed.2021.736495 (PMC8591138; doi:10.3389/fmed.2021.736495)
Supplement: Supplementary file 1 [file Data_Sheet_1.docx]

**Supplementary Materials**

**Identification of cross-pathway connections via protein-protein interactions linked to altered states of metabolic enzymes in cervical cancer.**

**Krishna Kumar*, Sarpita Bose and Saikat Chakrabarti***

**Structural Biology and Bioinformatics Division,**

**CSIR-Indian Institute of Chemical Biology,**

**Kolkata, WB, India**

***Author for correspondence**

**Email:** [**krishna.kumarbt2009@gmail.com**](mailto:krishna.kumarbt2009@gmail.com)**, saikat@iicb.res.in**

**
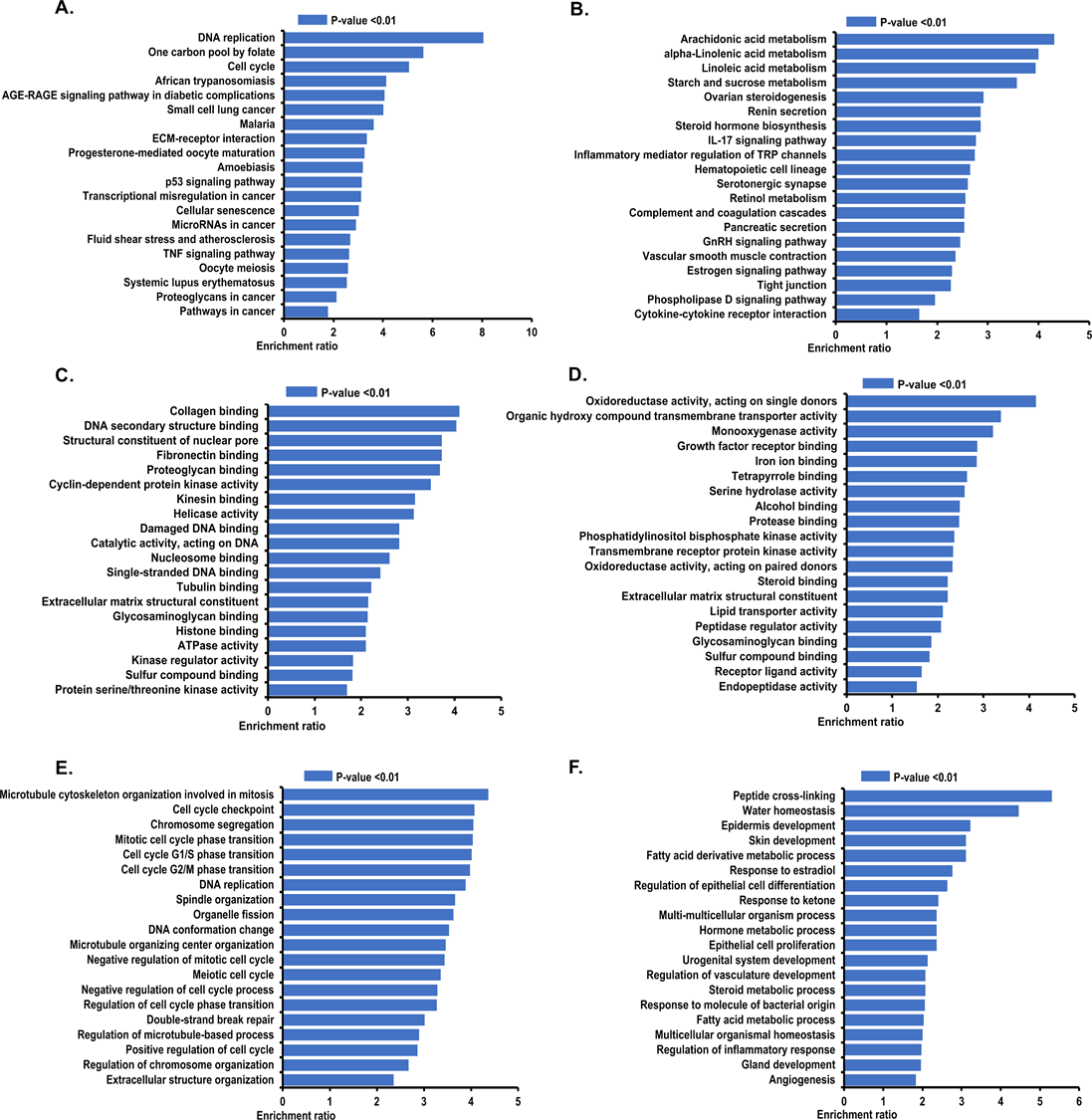
**

**Figure S1:** **Over-representation analysis (ORA) of deregulated genes in cervical cancer.** Panels A and B show enriched KEGG pathways, C and D show molecular function, E and F show biological process for up-regulated and down-regulated genes, respectively.

**
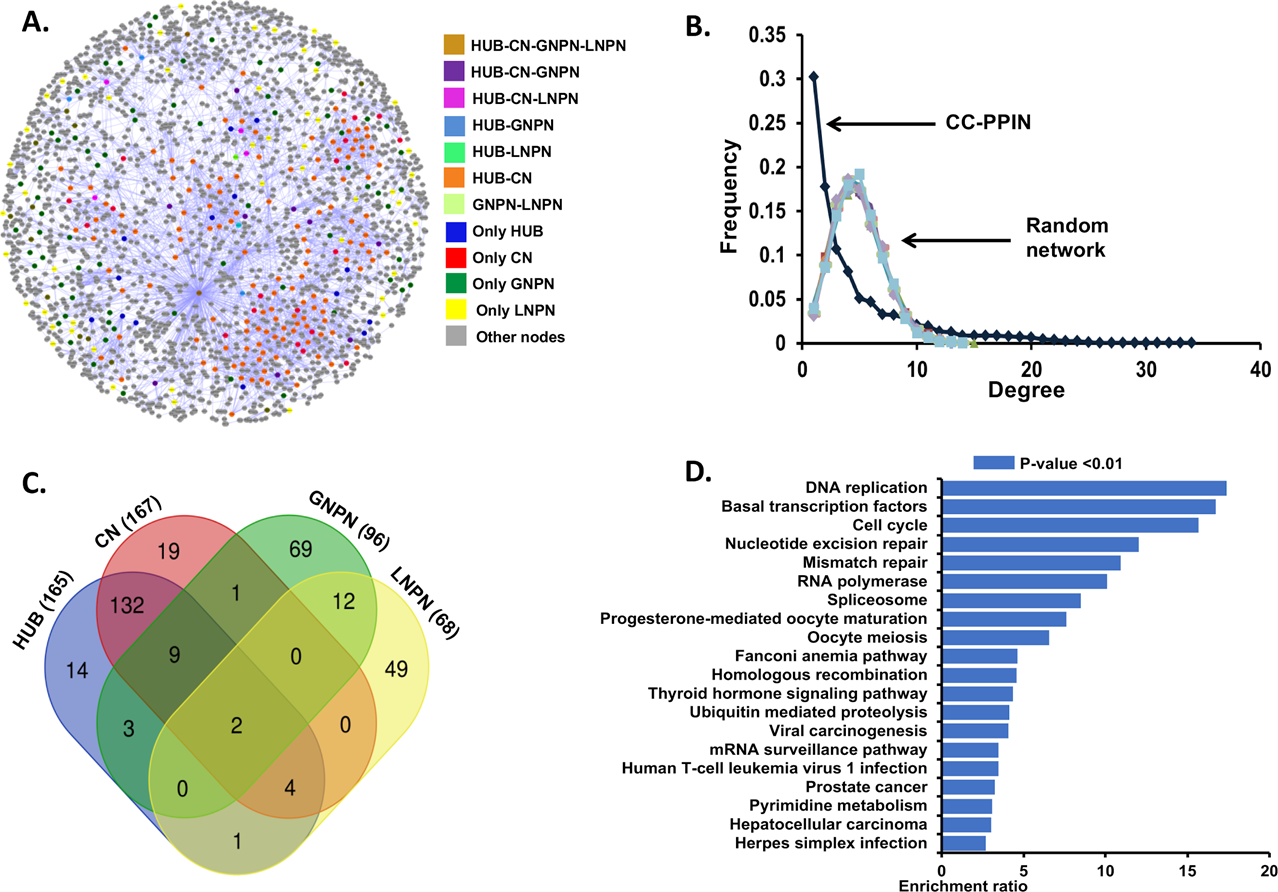
**

**Figure S2:** **Signaling-metabolic interaction network analysis in cervical cancer.** Topologically Important interacting nodes/proteins (IINs/IIPs) in cervical cancer specific protein-protein interaction network (CC-PPIN). Panel A represents CC-PPIN network. Nodes are colored by IIPs. Panel B is showing the degree distribution of CC-PPIN and 10 random networks. Random networks follow the binomial degree distribution whereas CC-PPIN follows the power law degree distribution. Panel C is showing the overlap among different network properties (HUB, CN, GNPN and LNPN). Panel D represents the KEGG pathways enrichment of IIPs.

**
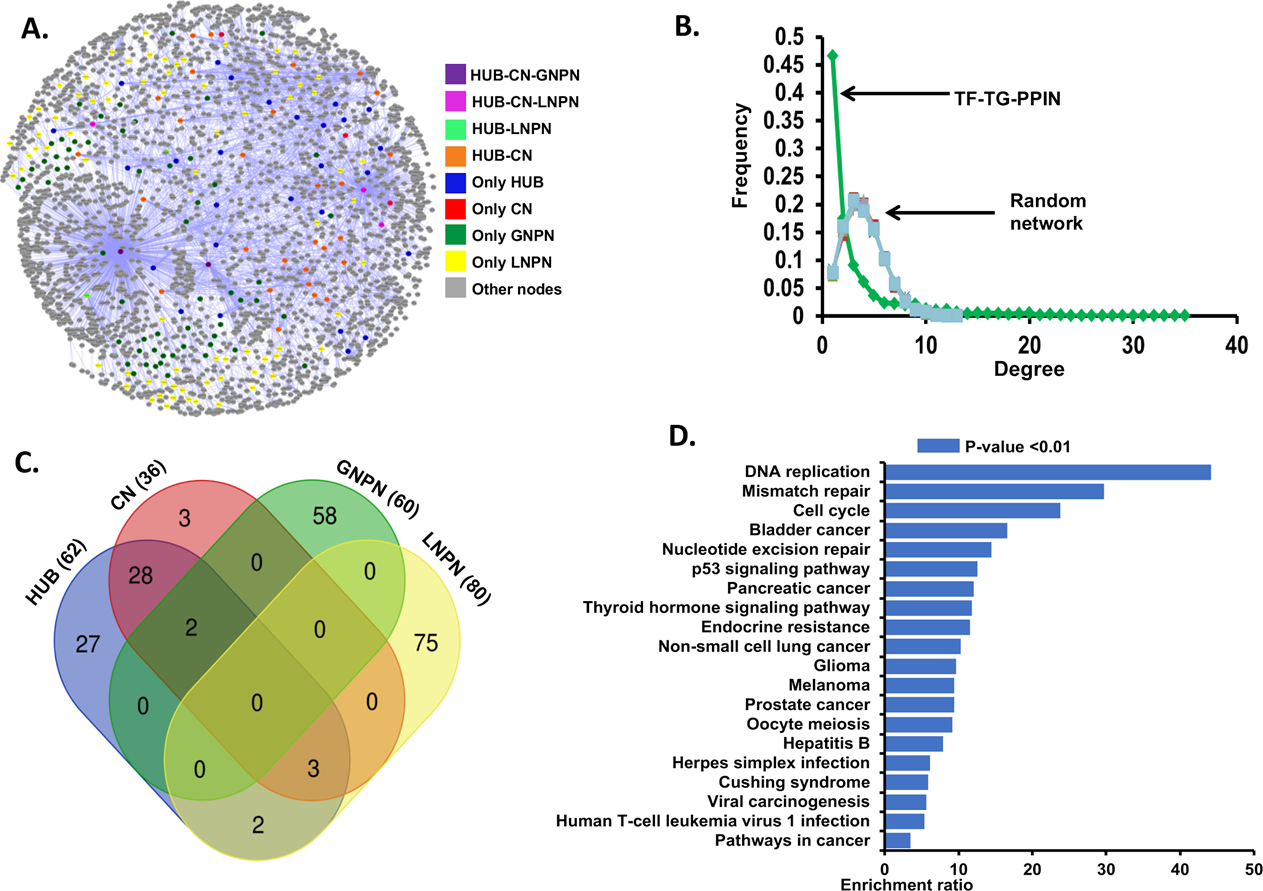
**

**Figure S3:** **Transcription factor-metabolic interaction network analysis in cervical cancer.** Topologically Important interacting nodes/proteins (IINs/IIPs) in cervical cancer specific TF-TG-PPIN. Panel A represents TF-TG-PPIN network. Nodes are colored by IIPs. Panel B is showing the degree distribution of TF-TG-PPIN and 10 random networks. Random networks follow the binomial degree distribution whereas CC-PPIN follows the power law degree distribution. Panel C is showing the overlap among different network properties (HUB, CN, GNPN and LNPN). Panel D represents the KEGG pathway enrichment of IINs.

**
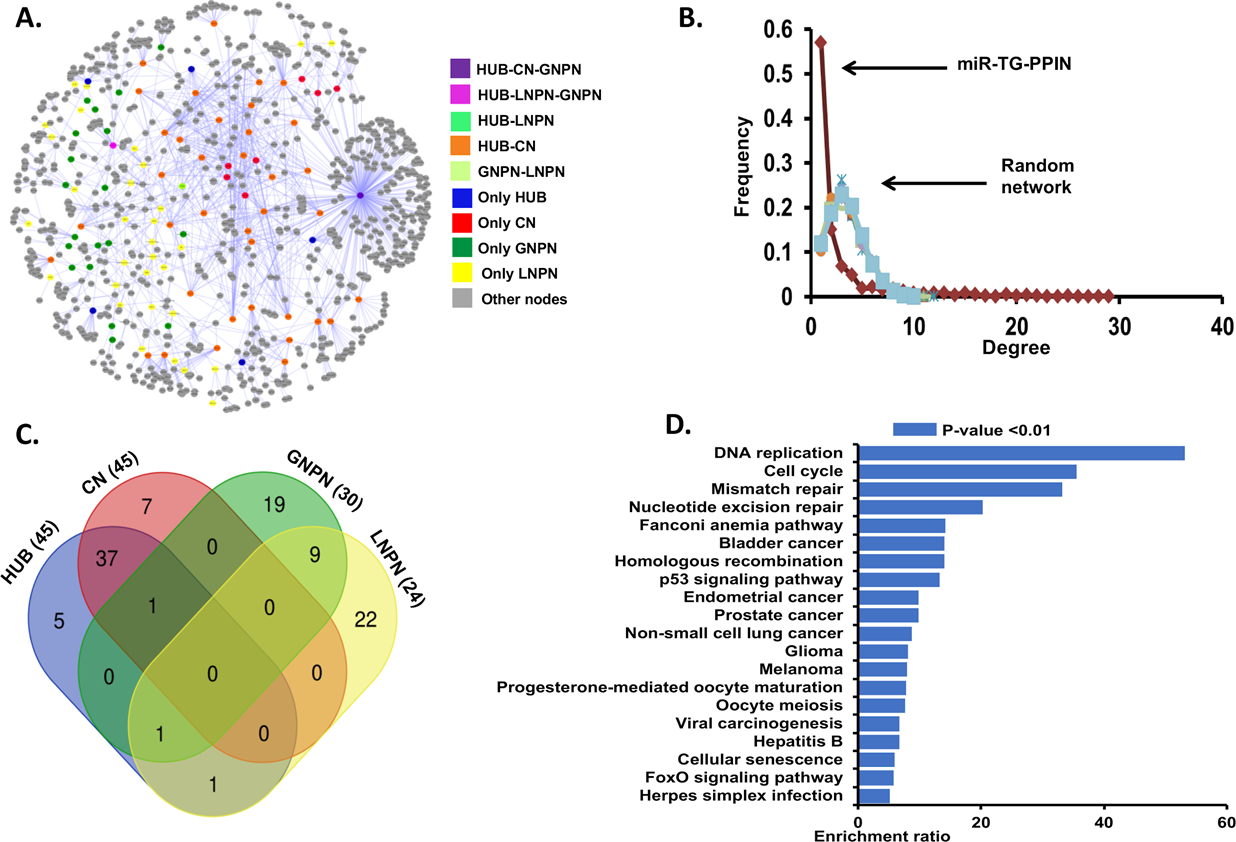
**

**Figure S4:** **miRNA-metabolic interaction network analysis in cervical cancer.** Topologically Important interacting nodes/proteins (IINs/IIPs) in cervical cancer specific miR-TG-PPIN. Panel A represents miR-TG-PPIN network. Nodes are colored by IINs. Panel B is showing the degree distribution of miR-TG-PPIN and 10 random networks. Random networks follow the binomial degree distribution whereas miR-TG-PPIN follows the power law degree distribution. Panel C is showing the overlap among different network properties (HUB, CN, GNPN and LNPN). Panel D represents the KEGG pathway enrichment of IINs.


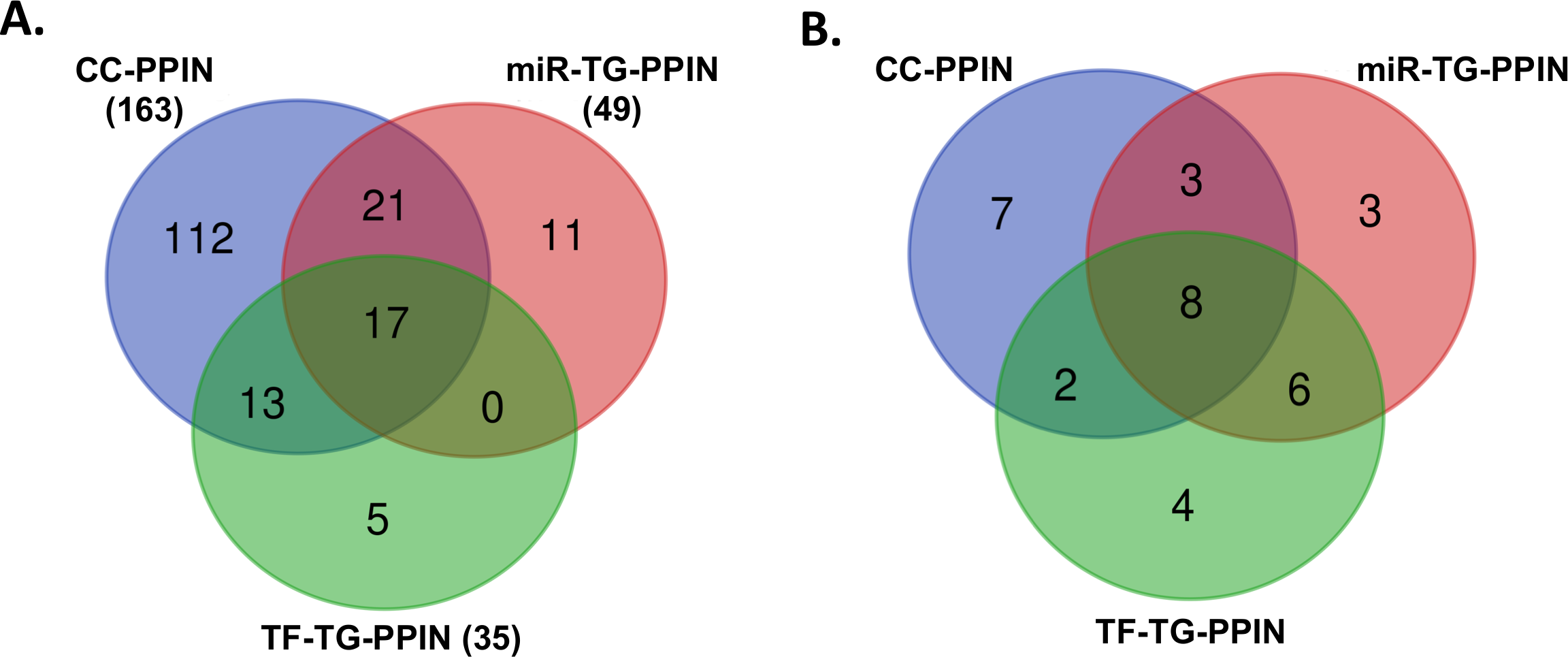


**Figure S5:** **Important interacting node (IIN) and enriched pathways comparison.** Panel A and B show the overlap of topologically Important interacting node (IINs) and overlap of top 20 enriched pathways for IINs, respectively, in cervical cancer specific PPIN, TF-TG-PPIN and miR-TG-PPIN.


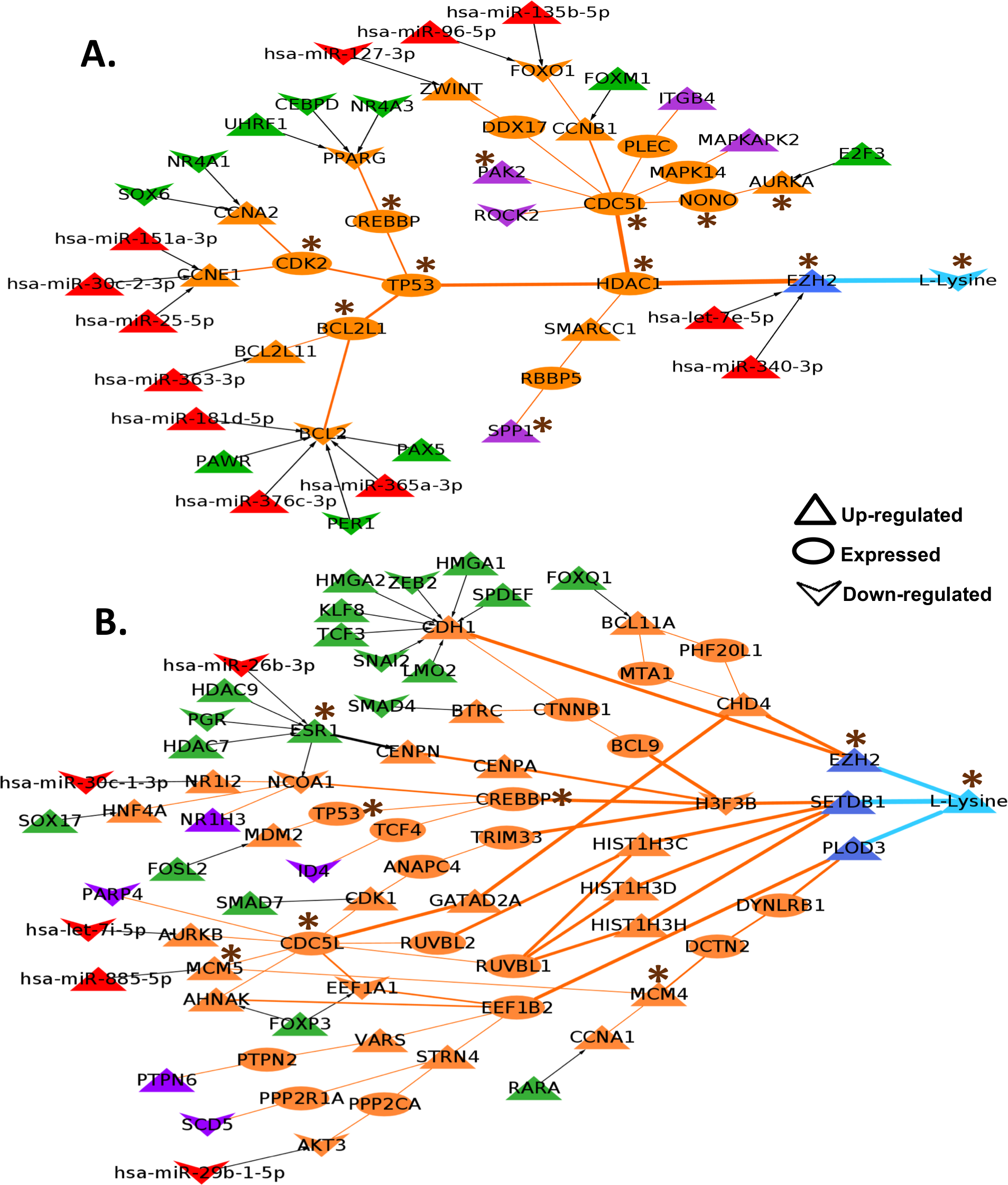


**Figure S6: Signaling pathway proteins, transcription factor and microRNA cross-connecting paths/links to L-lysine.** Panels A and B show the L-lysine connecting paths in breast and ovarian cancers, respectively. Terminal signaling pathway proteins, transcription factor and miRNAs, metabolic enzymes are colored in purple, green, red and blue. Protein-protein interactors are colored in orange. Gene regulatory edges are represented as black arrow and protein-protein interactions are represented by orange edges. Nodes with asterisk (*) are the nodes common in the significant paths/networks of cervical cancer.

**Table S1: IINs in cervical cancer specific protein-protein interaction network (CC-PPIN)**

**Table S2: IINs in cervical cancer specific transcriptional regulatory network (TF-TG-PPIN)**

**Table S3: IINs in cervical cancer specific post-transcriptional regulatory network (miR-TG-PPIN)**

**Table S4: Common IINs in cervical cancer specific PPIN, TF-TG-PPIN and miR-TG-PPIN**

**Table S5: Signaling to metabolic pathways interconnections**

**Table S6: Transcription factor to metabolic pathways interconnections**

**Table S7: microRNA to metabolic pathways interconnections**

**Table S8: Signaling to metabolic enzyme cross-connecting paths/links**

AKT3 GSK3B TRAF2 RIPK1 NDUFA11

ATP6V1C2 ATP6V1A USP5 TXNDC17 TYMS

BAD BCL2L1 CASP8 RIPK1 NDUFA11

BAD BCL2L1 TP53 FZR1 RRM2

BAD BCL2L1 TP53 HDAC1 EZH2

CD14 NDUFA11

CDKN2C CDK4 CCNA2 ANAPC4 RRM2

CDKN2C CDK4 CCNL1 MCM3 POLA1

CDKN2C CDK4 CDKN1A PCNA POLE

CFTR FLNA ERBB3 PIK3R1 PIK3CA

CFTR FLNA TRAF2 RIPK1 NDUFA11

CHEK1 CDC5L ACTB CFL2 AKR1B1

CHEK1 CDC5L ATP6AP2 ATP6V0A2

CHEK1 CDC5L BUB3 ANAPC4 RRM2

CHEK1 CDC5L CAPG ADK NQO1

CHEK1 CDC5L CAPG MIF AKR1C2

CHEK1 CDC5L CCNB1 ANAPC4 RRM2

CHEK1 CDC5L CDK1 ANAPC4 RRM2

CHEK1 CDC5L CSE1L ATP6V1A ATP6V1C2

CHEK1 CDC5L DDX3X IMPDH1 GMPS

CHEK1 CDC5L EEF1A1 EEF1G PLOD3

CHEK1 CDC5L HDAC1 EZH2

CHEK1 CDC5L HSPA5 DNAJB11 RRM1

CHEK1 CDC5L MAPK14 OBSL1 ALDH3B2

CHEK1 CDC5L MCM4 MCM3 POLA1

CHEK1 CDC5L MCM5 MCM3 POLA1

CHEK1 CDC5L RAD18 PCNA POLE

CHEK1 CDC5L RFC3 MCM3 POLA1

CHEK1 CDC5L RFC4 MCM3 POLA1

CHEK1 CDC5L RUVBL2 PCNA POLE

CHEK1 CDC5L SCYL2 COX17

CHEK1 CDC5L SEPT9 SUCLG1 SUCLG2

EREG EGFR PIK3R1 PIK3CA

ESR1 FN1 EGFR PIK3R1 PIK3CA

FGFR2 PLCG1 EGFR PIK3R1 PIK3CA

FGFR2 PLCG1 MCMBP MCM3 POLA1

GNAS EIF4H KDM3A GART

GNB5 CCT5 CDC20 ANAPC4 RRM2

GNB5 CCT5 CDC20 PCNA POLE

GNB5 CCT5 CDC5L ATP6AP2 ATP6V0A2

GNB5 CCT5 CDC5L HDAC1 EZH2

GNB5 CCT5 CDC5L SCYL2 COX17

IL1R1 IRAK1 TAB2 RIPK1 NDUFA11

MAML3 NOTCH4 CDC5L ATP6AP2 ATP6V0A2

MAML3 NOTCH4 CDC5L HDAC1 EZH2

MAML3 NOTCH4 CDC5L SCYL2 COX17

MAP3K1 TRAF2 HTRA2 NADK2 MPST

MAP3K1 TRAF2 RIPK1 NDUFA11

MAP3K1 TRAF2 RIPK1 NDUFA9 NDUFB4

PAK2 CDC5L ACTB CFL2 AKR1B1

PAK2 CDC5L ATP6AP2 ATP6V0A2

PAK2 CDC5L BUB3 ANAPC4 RRM2

PAK2 CDC5L CAPG ADK NQO1

PAK2 CDC5L CAPG MIF AKR1C2

PAK2 CDC5L CCNB1 ANAPC4 RRM2

PAK2 CDC5L CDK1 ANAPC4 RRM2

PAK2 CDC5L CSE1L ATP6V1A ATP6V1C2

PAK2 CDC5L DDX3X IMPDH1 GMPS

PAK2 CDC5L EEF1A1 EEF1G PLOD3

PAK2 CDC5L HDAC1 EZH2

PAK2 CDC5L HSPA5 DNAJB11 RRM1

PAK2 CDC5L MAPK14 OBSL1 ALDH3B2

PAK2 CDC5L MCM4 MCM3 POLA1

PAK2 CDC5L MCM5 MCM3 POLA1

PAK2 CDC5L RAD18 PCNA POLE

PAK2 CDC5L RFC3 MCM3 POLA1

PAK2 CDC5L RFC4 MCM3 POLA1

PAK2 CDC5L RUVBL2 PCNA POLE

PAK2 CDC5L SCYL2 COX17

PAK2 CDC5L SEPT9 SUCLG1 SUCLG2

PDGFRA PIK3R1 PIK3CA

PPARD MDM4 TP53 FZR1 RRM2

PPARD MDM4 TP53 HDAC1 EZH2

PTK2 ERBB2 PIK3R1 PIK3CA

RBL1 CCNA2 ANAPC4 RRM2

RBL1 CCNA2 CDK2 MCM3 POLA1

RBL1 CCNA2 CDKN1A PCNA POLE

RICTOR MTOR FKBP1A ENO2

RICTOR MTOR FKBP1A FABP5 AKR1B1

RICTOR MTOR IKBKG RIPK1 NDUFA11

RICTOR MTOR RRAGC PITRM1 SUCLG2

SERPINE1 VTN LUC7L3 ZRANB2 TYMS

SMC1B RAD21 MSH6 PCNA POLE

SPP1 RBBP5 CDC5L ATP6AP2 ATP6V0A2

SPP1 RBBP5 CDC5L HDAC1 EZH2

SPP1 RBBP5 CDC5L SCYL2 COX17

TF MIS12 BUB1B ANAPC4 RRM2

TF MIS12 NDC80 ATP6V1D ATP6V0A2

TGFA EGFR PIK3R1 PIK3CA

TNFRSF1B TNF RIPK1 NDUFA11

TNFRSF1B TNF RIPK1 NDUFA9 NDUFB4

TNFRSF1B TNF RIPK1 NDUFA9 NDUFS7

TRAF5 TBK1 TLR3 RIPK1 NDUFA11

TRAF5 TBK1 TRAF2 RIPK1 NDUFA11

**Table S9: Transcription factor to metabolic enzyme cross-connecting paths/links**

AR AKR1B1

AR AKR1B1 ENO2

AR AKR1C3 AKR1C1

AR AKR1C3 AKR1C2

AR CDA

AR COX17

AR DPYSL3 DPYS

AR EZH2

AR PIK3CA

AR POLA1

AR POLA1 POLE

AR POLA1 POLE POLE2

AR POLA2

AR PRIM1

E2F1 CYP27B1

E2F1 DHFR

E2F1 KIF2C CDC5L ATP6AP2 ATP6V0A2

E2F1 KIF2C CDC5L HDAC1 EZH2

E2F1 RRM1

E2F1 TYMS

E2F1 TYMS UFC1 COX17

EGR1 CCND1 CDKN1A PCNA POLE

EGR1 FN1 AURKA SEPT9 SUCLG1 SUCLG2

EGR1 FN1 EGFR PIK3R1 PIK3CA

EGR1 TMPO CDC5L ACTB CFL2 AKR1B1

EGR1 TMPO CDC5L AHNAK EEF1B2 PLOD3

EGR1 TMPO CDC5L ATP6AP2 ATP6V0A2

EGR1 TMPO CDC5L DDX3X IMPDH1 GMPS

EGR1 TMPO CDC5L HDAC1 EZH2

EGR1 TMPO CDC5L HSPA5 DNAJB11 RRM1

EGR1 TMPO CDC5L RAD18 PCNA POLE

EGR1 TMPO CDC5L SCYL2 COX17

ESR1 FANCI CDC5L AHNAK EEF1B2 PLOD3

ESR1 FANCI CDC5L ATP6AP2 ATP6V0A2

ESR1 FANCI CDC5L HSPA5 DNAJB11 RRM1

ESR1 NASP RBBP4 HDAC1 EZH2

ESR1 NQO1

ESR1 POLE

ESR1 POLE POLA1 POLA2

ESR1 POLE POLA1 PRIM1

ESR1 TGFA EGFR PIK3R1 PIK3CA

ESR1 TYMS UFC1 COX17

FOSL1 PLAUR VMA21 ATP6AP2 ATP6V0A2

FOSL1 PLAUR VTN LUC7L3 ZRANB2 TYMS

FOXM1 CCNB1 ANAPC4 RRM2

FOXM1 CCNB1 CDC5L SCYL2 COX17

FOXM1 CDC6 MCM3 POLA1

FOXM1 CDC6 MCM3 POLA1 POLA2

FOXM1 CDC6 MCM3 POLA1 POLE

FOXM1 CDC6 MCM3 POLA1 POLE POLE2

FOXM1 CDC6 MCM3 POLA1 PRIM1

GATA3 CDKN2A CDC45 MCM3 POLA1

GATA3 CDKN2A CDC45 MCM3 POLA1 POLA2

GATA3 CDKN2A CDC45 MCM3 POLA1 PRIM1

GATA3 CDKN2A CDC45 PCNA POLE

GATA3 CDKN2A CDC7 MCM3 POLA1

GATA3 CDKN2A CDC7 MCM3 POLA1 POLA2

GATA3 CDKN2A CDC7 MCM3 POLA1 PRIM1

GATA3 CDKN2A MCM5 CDC5L SCYL2 COX17

GATA3 CDKN2A ORC5 CDC5L SCYL2 COX17

HSF2 HSPA4 HSPA8 CDC5L HDAC1 EZH2

HSF2 HSPA4 HSPA8 CDC5L SCYL2 COX17

HSF2 HSPA4 HSPA8 SF3A2 ATP6AP2 ATP6V0A2

HSF2 HSPA4 HSPA8 SF3A2 SUGP1 ACAA1

ID4 BRCA1 MRE11A CDC5L ATP6AP2 ATP6V0A2

ID4 BRCA1 MRE11A CDC5L HDAC1 EZH2

ID4 BRCA1 MRE11A CDC5L SCYL2 COX17

ID4 BRCA1 RAD50 CDC5L ATP6AP2 ATP6V0A2

ID4 BRCA1 RAD50 CDC5L HDAC1 EZH2

ID4 BRCA1 RAD50 CDC5L SCYL2 COX17

ING1 BAX BCL2L1 TP53 HDAC1 EZH2

ING1 BAX BID CASP8 RIPK1 NDUFA11

ING1 BAX PRDX1 NANS FAHD1

ING1 BAX PRDX2 PARK7 MIF AKR1C2

ING1 BAX PRDX2 PARK7 MIF COX17

IRF6 CDKN2A CDC45 MCM3 POLA1

IRF6 CDKN2A CDC45 MCM3 POLA1 POLA2

IRF6 CDKN2A CDC45 MCM3 POLA1 PRIM1

IRF6 CDKN2A CDC45 PCNA POLE

IRF6 CDKN2A CDC7 MCM3 POLA1

IRF6 CDKN2A CDC7 MCM3 POLA1 POLA2

IRF6 CDKN2A CDC7 MCM3 POLA1 PRIM1

IRF6 CDKN2A MCM5 CDC5L SCYL2 COX17

IRF6 CDKN2A ORC5 CDC5L SCYL2 COX17

KAT2B CDK2 AURKA SEPT9 SUCLG1 SUCLG2

KAT2B CDK2 CCNB1 ANAPC4 RRM2

KAT2B CDK2 CCNB1 CDC5L SCYL2 COX17

KAT2B CDK2 CDK1 ANAPC4 RRM2

KAT2B CDK2 CDK1 CDC5L SCYL2 COX17

KAT2B CDK2 MCM3 POLA1

KAT2B CDK2 MCM3 POLA1 POLA2

KAT2B CDK2 MCM3 POLA1 POLE

KAT2B CDK2 MCM3 POLA1 POLE POLE2

KAT2B CDK2 MCM3 POLA1 PRIM1

KAT2B CDK2 TP53 HDAC1 EZH2

KLF8 FN1 AURKA CDK1 ANAPC4 RRM2

KLF8 FN1 AURKA CDK1 PCNA POLE

KLF8 FN1 AURKA CDK2 MCM3 POLA1

KLF8 FN1 AURKA SEPT9 SUCLG1 SUCLG2

KLF8 FN1 EGFR PIK3R1 PIK3CA

LHX2 CDKN2A CDC45 MCM3 POLA1

LHX2 CDKN2A CDC45 MCM3 POLA1 POLA2

LHX2 CDKN2A CDC45 MCM3 POLA1 PRIM1

LHX2 CDKN2A CDC45 PCNA POLE

LHX2 CDKN2A CDC7 MCM3 POLA1

LHX2 CDKN2A CDC7 MCM3 POLA1 POLA2

LHX2 CDKN2A CDC7 MCM3 POLA1 PRIM1

LHX2 CDKN2A MCM5 CDC5L SCYL2 COX17

LHX2 CDKN2A ORC5 CDC5L SCYL2 COX17

MED1 AURKA CDK1 ANAPC4 RRM2

MED1 AURKA CDK1 PCNA POLE

MED1 AURKA CDK2 MCM3 POLA1 POLA2

MED1 AURKA CDK2 MCM3 POLA1 PRIM1

MED1 AURKA DDX17 TXLNA IARS HMGCS1

MED1 AURKA DDX17 TXLNA KARS HMGCS1

MED1 AURKA FN1 EGFR PIK3R1 PIK3CA

MED1 AURKA FUS CDC5L HDAC1 EZH2

MED1 AURKA FUS CDC5L SCYL2 COX17

MED1 AURKA FUS SF3A2 ATP6AP2 ATP6V0A2

MED1 AURKA FUS SF3A2 SUGP1 ACAA1

MED1 AURKA NONO CDC5L HDAC1 EZH2

MED1 AURKA NONO CDC5L SCYL2 COX17

MED1 AURKA SEPT9 SEPT11 SEC22B TST

MED1 AURKA SEPT9 SEPT11 TMEM189 MPST

MED1 AURKA SEPT9 SUCLG1 SUCLG2

MEOX2 CDKN2A CDC45 MCM3 POLA1

MEOX2 CDKN2A CDC45 MCM3 POLA1 POLA2

MEOX2 CDKN2A CDC45 MCM3 POLA1 PRIM1

MEOX2 CDKN2A CDC45 PCNA POLE

MEOX2 CDKN2A CDC7 MCM3 POLA1

MEOX2 CDKN2A CDC7 MCM3 POLA1 POLA2

MEOX2 CDKN2A CDC7 MCM3 POLA1 PRIM1

MEOX2 CDKN2A MCM5 CDC5L SCYL2 COX17

MEOX2 CDKN2A ORC5 CDC5L SCYL2 COX17

NCOA2 AR BUD31 CDC5L HDAC1 EZH2

NCOA2 AR BUD31 CDC5L SCYL2 COX17

NCOA2 AR BUD31 SF3A2 ATP6AP2 ATP6V0A2

NCOA2 AR BUD31 SF3A2 SUGP1 ACAA1

PGR HLTF CDC5L SCYL2 COX17

PGR HLTF S100A10 AHNAK EEF1B2 PLOD3

PLAGL1 CDKN2A CDC45 MCM3 POLA1

PLAGL1 CDKN2A CDC45 MCM3 POLA1 POLA2

PLAGL1 CDKN2A CDC45 MCM3 POLA1 PRIM1

PLAGL1 CDKN2A CDC45 PCNA POLE

PLAGL1 CDKN2A CDC7 MCM3 POLA1

PLAGL1 CDKN2A CDC7 MCM3 POLA1 POLA2

PLAGL1 CDKN2A CDC7 MCM3 POLA1 PRIM1

PLAGL1 CDKN2A MCM5 CDC5L SCYL2 COX17

PLAGL1 CDKN2A ORC5 CDC5L SCYL2 COX17

RBL1 BRCA1 MRE11A CDC5L ATP6AP2 ATP6V0A2

RBL1 BRCA1 MRE11A CDC5L HDAC1 EZH2

RBL1 BRCA1 MRE11A CDC5L SCYL2 COX17

RBL1 BRCA1 RAD50 CDC5L ATP6AP2 ATP6V0A2

RBL1 BRCA1 RAD50 CDC5L HDAC1 EZH2

RBL1 BRCA1 RAD50 CDC5L SCYL2 COX17

TBX3 CDKN2A CDC45 MCM3 POLA1

TBX3 CDKN2A CDC45 MCM3 POLA1 POLA2

TBX3 CDKN2A CDC45 MCM3 POLA1 PRIM1

TBX3 CDKN2A CDC45 PCNA POLE

TBX3 CDKN2A CDC7 MCM3 POLA1

TBX3 CDKN2A CDC7 MCM3 POLA1 POLA2

TBX3 CDKN2A CDC7 MCM3 POLA1 PRIM1

TBX3 CDKN2A MCM5 CDC5L SCYL2 COX17

TBX3 CDKN2A ORC5 CDC5L SCYL2 COX17

TGIF1 SERPINE1 VTN LUC7L3 ZRANB2 TYMS

TWIST1 AKR1C2 AKR1C1

TWIST1 AKR1C2 MIF COX17

TWIST1 DNM3 DNM2 CDC5L ATP6AP2 ATP6V0A2

TWIST1 DNM3 DNM2 CDC5L HDAC1 EZH2

WHSC1 CDKN2A CDC45 MCM3 POLA1

WHSC1 CDKN2A CDC45 MCM3 POLA1 POLA2

WHSC1 CDKN2A CDC45 MCM3 POLA1 PRIM1

WHSC1 CDKN2A CDC45 PCNA POLE

WHSC1 CDKN2A CDC7 MCM3 POLA1

WHSC1 CDKN2A CDC7 MCM3 POLA1 POLA2

WHSC1 CDKN2A CDC7 MCM3 POLA1 PRIM1

WHSC1 CDKN2A MCM5 CDC5L SCYL2 COX17

WHSC1 CDKN2A ORC5 CDC5L SCYL2 COX17

ZEB1 AR BUD31 CDC5L HDAC1 EZH2

ZEB1 AR BUD31 CDC5L SCYL2 COX17

ZEB1 AR BUD31 SF3A2 ATP6AP2 ATP6V0A2

ZEB1 AR BUD31 SF3A2 SUGP1 ACAA1

ZIC2 CDKN2A CDC45 MCM3 POLA1

ZIC2 CDKN2A CDC45 MCM3 POLA1 POLA2

ZIC2 CDKN2A CDC45 MCM3 POLA1 PRIM1

ZIC2 CDKN2A CDC45 PCNA POLE

ZIC2 CDKN2A CDC7 MCM3 POLA1

ZIC2 CDKN2A CDC7 MCM3 POLA1 POLA2

ZIC2 CDKN2A CDC7 MCM3 POLA1 PRIM1

ZIC2 CDKN2A MCM5 CDC5L SCYL2 COX17

ZIC2 CDKN2A ORC5 CDC5L SCYL2 COX17

ZNF134 CDKN2A CDC45 MCM3 POLA1

ZNF134 CDKN2A CDC45 MCM3 POLA1 POLA2

ZNF134 CDKN2A CDC45 MCM3 POLA1 PRIM1

ZNF134 CDKN2A CDC45 PCNA POLE

ZNF134 CDKN2A CDC7 MCM3 POLA1

ZNF134 CDKN2A CDC7 MCM3 POLA1 POLA2

ZNF134 CDKN2A CDC7 MCM3 POLA1 PRIM1

ZNF134 CDKN2A MCM5 CDC5L SCYL2 COX17

ZNF134 CDKN2A ORC5 CDC5L SCYL2 COX17

ZNF135 CDKN2A CDC45 MCM3 POLA1

ZNF135 CDKN2A CDC45 MCM3 POLA1 POLA2

ZNF135 CDKN2A CDC45 MCM3 POLA1 PRIM1

ZNF135 CDKN2A CDC45 PCNA POLE

ZNF135 CDKN2A CDC7 MCM3 POLA1

ZNF135 CDKN2A CDC7 MCM3 POLA1 POLA2

ZNF135 CDKN2A CDC7 MCM3 POLA1 PRIM1

ZNF135 CDKN2A MCM5 CDC5L SCYL2 COX17

ZNF135 CDKN2A ORC5 CDC5L SCYL2 COX17

ZNF419 CDKN2A CDC45 MCM3 POLA1

ZNF419 CDKN2A CDC45 MCM3 POLA1 POLA2

ZNF419 CDKN2A CDC45 MCM3 POLA1 PRIM1

ZNF419 CDKN2A CDC45 PCNA POLE

ZNF419 CDKN2A CDC7 MCM3 POLA1

ZNF419 CDKN2A CDC7 MCM3 POLA1 POLA2

ZNF419 CDKN2A CDC7 MCM3 POLA1 PRIM1

ZNF419 CDKN2A MCM5 CDC5L SCYL2 COX17

ZNF419 CDKN2A ORC5 CDC5L SCYL2 COX17

ZSCAN18 CDKN2A CDC45 MCM3 POLA1

ZSCAN18 CDKN2A CDC45 MCM3 POLA1 POLA2

ZSCAN18 CDKN2A CDC45 MCM3 POLA1 PRIM1

ZSCAN18 CDKN2A CDC45 PCNA POLE

ZSCAN18 CDKN2A CDC7 MCM3 POLA1

ZSCAN18 CDKN2A CDC7 MCM3 POLA1 POLA2

ZSCAN18 CDKN2A CDC7 MCM3 POLA1 PRIM1

ZSCAN18 CDKN2A MCM5 CDC5L SCYL2 COX17

ZSCAN18 CDKN2A ORC5 CDC5L SCYL2 COX17

ZXDC CDKN2A CDC45 MCM3 POLA1

ZXDC CDKN2A CDC45 MCM3 POLA1 POLA2

ZXDC CDKN2A CDC45 MCM3 POLA1 PRIM1

ZXDC CDKN2A CDC45 PCNA POLE

ZXDC CDKN2A CDC7 MCM3 POLA1

ZXDC CDKN2A CDC7 MCM3 POLA1 POLA2

ZXDC CDKN2A CDC7 MCM3 POLA1 PRIM1

ZXDC CDKN2A MCM5 CDC5L SCYL2 COX17

ZXDC CDKN2A ORC5 CDC5L SCYL2 COX17

**Table S10: miRNA to metabolic enzyme cross-connecting paths/links**

hsa-miR-106b-5p RBL1 CCNA2 ANAPC4 RRM2

hsa-miR-138-1-3p PDK1 MTOR FKBP1A ENO2

hsa-miR-138-1-3p PDK1 MTOR IKBKG RIPK1 NDUFA11

hsa-miR-138-5p EZH2

hsa-miR-147a MCM3 CDC6 CCNA2 ANAPC4 RRM2

hsa-miR-147a MCM3 CDK2 CCNB1 ANAPC4 RRM2

hsa-miR-147a MCM3 MCM4 CDC5L HDAC1 EZH2

hsa-miR-147a MCM3 MCM5 CDC5L HDAC1 EZH2

hsa-miR-147a MCM3 POLA1 POLE4 POLE2

hsa-miR-147a MCM3 POLA1 PRIM1

hsa-miR-147a MCM3 RFC3 CDC5L HDAC1 EZH2

hsa-miR-147a MCM3 RFC4 CDC5L HDAC1 EZH2

hsa-miR-15b-5p CHEK1 CDC5L BUB3 ANAPC4 RRM2

hsa-miR-15b-5p CHEK1 CDC5L HDAC1 EZH2

hsa-miR-16-5p CHEK1 CDC5L HDAC1 EZH2

hsa-miR-193b-3p AKR1C2 AKR1C3

hsa-miR-196a-5p TYMS

hsa-miR-203a-3p STAT1 CREBBP TP53 HDAC1 EZH2

hsa-miR-223-3p CDK2 TP53 HDAC1 EZH2

hsa-miR-223-3p CFTR FLNA TRAF2 RIPK1 NDUFA11

hsa-miR-223-3p STAT1 CREBBP TP53 HDAC1 EZH2

hsa-miR-494-3p CFTR FLNA TRAF2 RIPK1 NDUFA11

hsa-miR-593-5p PLK1 CDC20 PCNA POLE POLE2

hsa-miR-593-5p PLK1 CDC5L CAPG MIF AKR1C2

hsa-miR-593-5p PLK1 CDC5L HDAC1 EZH2

hsa-miR-593-5p PLK1 CDC5L MAPK14 OBSL1 ALDH3B2

hsa-miR-593-5p PLK1 FZR1 RRM2

**Table S11: Signaling-metabolic, TF-metabolic and miRNA-metabolic commonly connecting paths/links**

AKT3 GSK3B TRAF2 RIPK1 NDUFA11

AR AKR1B1 ENO2

AR AKR1C3 AKR1C2

AR EZH2

ATP6V1C2 ATP6V1A USP5 TXNDC17 TYMS

BAD BCL2L1 CASP8 RIPK1 NDUFA11

BAD BCL2L1 TP53 FZR1 RRM2

BAD BCL2L1 TP53 HDAC1 EZH2

CD14 NDUFA11

CDKN2C CDK4 CCNA2 ANAPC4 RRM2

CFTR FLNA TRAF2 RIPK1 NDUFA11

CHEK1 CDC5L BUB3 ANAPC4 RRM2

CHEK1 CDC5L CAPG MIF AKR1C2

CHEK1 CDC5L CCNB1 ANAPC4 RRM2

CHEK1 CDC5L CDK1 ANAPC4 RRM2

CHEK1 CDC5L HDAC1 EZH2

E2F1 KIF2C CDC5L HDAC1 EZH2

E2F1 TYMS

E2F1 TYMS UFC1 COX17

EGR1 TMPO CDC5L HDAC1 EZH2

ESR1 NASP RBBP4 HDAC1 EZH2

FOSL1 PLAUR VTN LUC7L3 ZRANB2 TYMS

FOXM1 CCNB1 ANAPC4 RRM2

GNB5 CCT5 CDC20 ANAPC4 RRM2

GNB5 CCT5 CDC5L HDAC1 EZH2

hsa-miR-106b-5p RBL1 CCNA2 ANAPC4 RRM2

hsa-miR-138-1-3p PDK1 MTOR FKBP1A ENO2

hsa-miR-138-1-3p PDK1 MTOR IKBKG RIPK1 NDUFA11

hsa-miR-138-5p EZH2

hsa-miR-147a MCM3 CDC6 CCNA2 ANAPC4 RRM2

hsa-miR-147a MCM3 CDK2 CCNB1 ANAPC4 RRM2

hsa-miR-147a MCM3 MCM4 CDC5L HDAC1 EZH2

hsa-miR-147a MCM3 MCM5 CDC5L HDAC1 EZH2

hsa-miR-147a MCM3 RFC3 CDC5L HDAC1 EZH2

hsa-miR-147a MCM3 RFC4 CDC5L HDAC1 EZH2

hsa-miR-15b-5p CHEK1 CDC5L BUB3 ANAPC4 RRM2

hsa-miR-15b-5p CHEK1 CDC5L HDAC1 EZH2

hsa-miR-16-5p CHEK1 CDC5L HDAC1 EZH2

hsa-miR-193b-3p AKR1C2 AKR1C3

hsa-miR-196a-5p TYMS

hsa-miR-203a-3p STAT1 CREBBP TP53 HDAC1 EZH2

hsa-miR-223-3p CDK2 TP53 HDAC1 EZH2

hsa-miR-223-3p CFTR FLNA TRAF2 RIPK1 NDUFA11

hsa-miR-223-3p STAT1 CREBBP TP53 HDAC1 EZH2

hsa-miR-494-3p CFTR FLNA TRAF2 RIPK1 NDUFA11

hsa-miR-593-5p PLK1 CDC5L CAPG MIF AKR1C2

hsa-miR-593-5p PLK1 CDC5L HDAC1 EZH2

hsa-miR-593-5p PLK1 FZR1 RRM2

HSF2 HSPA4 HSPA8 CDC5L HDAC1 EZH2

ID4 BRCA1 MRE11A CDC5L HDAC1 EZH2

ID4 BRCA1 RAD50 CDC5L HDAC1 EZH2

IL1R1 IRAK1 TAB2 RIPK1 NDUFA11

ING1 BAX BCL2L1 TP53 HDAC1 EZH2

ING1 BAX BID CASP8 RIPK1 NDUFA11

ING1 BAX PRDX2 PARK7 MIF AKR1C2

KAT2B CDK2 CCNB1 ANAPC4 RRM2

KAT2B CDK2 CDK1 ANAPC4 RRM2

KAT2B CDK2 TP53 HDAC1 EZH2

KLF8 FN1 AURKA CDK1 ANAPC4 RRM2

MAML3 NOTCH4 CDC5L HDAC1 EZH2

MAP3K1 TRAF2 RIPK1 NDUFA11

MED1 AURKA CDK1 ANAPC4 RRM2

MED1 AURKA FUS CDC5L HDAC1 EZH2

MED1 AURKA NONO CDC5L HDAC1 EZH2

NCOA2 AR BUD31 CDC5L HDAC1 EZH2

PAK2 CDC5L BUB3 ANAPC4 RRM2

PAK2 CDC5L CAPG MIF AKR1C2

PAK2 CDC5L CCNB1 ANAPC4 RRM2

PAK2 CDC5L CDK1 ANAPC4 RRM2

PAK2 CDC5L HDAC1 EZH2

PPARD MDM4 TP53 FZR1 RRM2

PPARD MDM4 TP53 HDAC1 EZH2

RBL1 BRCA1 MRE11A CDC5L HDAC1 EZH2

RBL1 BRCA1 RAD50 CDC5L HDAC1 EZH2

RBL1 CCNA2 ANAPC4 RRM2

RICTOR MTOR FKBP1A ENO2

RICTOR MTOR IKBKG RIPK1 NDUFA11

SERPINE1 VTN LUC7L3 ZRANB2 TYMS

SPP1 RBBP5 CDC5L HDAC1 EZH2

TF MIS12 BUB1B ANAPC4 RRM2

TGIF1 SERPINE1 VTN LUC7L3 ZRANB2 TYMS

TNFRSF1B TNF RIPK1 NDUFA11

TRAF5 TBK1 TLR3 RIPK1 NDUFA11

TRAF5 TBK1 TRAF2 RIPK1 NDUFA11

TWIST1 AKR1C2 AKR1C1

TWIST1 AKR1C2 MIF COX17

TWIST1 DNM3 DNM2 CDC5L HDAC1 EZH2

ZEB1 AR BUD31 CDC5L HDAC1 EZH2

**Table S12: Genes/miRNAs in Signaling-metabolic, TF-metabolic and miRNA-metabolic commonly connecting paths/links with prognostic value in cervical cancer**

Provided as separate excel (Table S12.xlsx) file.

**Table S13: Response to chemotherapy (ROC plotter)**

| **Gene Symbol** | **AUC** | **ROC p-value** | **Median Expression** | |
| --- | --- | --- | --- | --- |
|  |  |  | **Responder** | **Non-responder** |
| BCL2L11 | 0.697 | 3.9e-10 | 471 | 226 |
| CCNE1 | 0.677 | 1.7e-08 | 388 | 254 |
| BCL2L1 | 0.674 | 3.8e-15 | 159 | 245 |
| ITGB4 | 0.674 | 2.5e-06 | 537 | 279 |
| DDX17 | 0.664 | 3.4e-06 | 5494 | 2595 |
| PAWR | 0.662 | 8.1e-06 | 738 | 256 |
| PLEC | 0.66 | 5.3e-13 | 164 | 240 |
| CDC5L | 0.656 | 3.9e-12 | 476 | 627 |
| TP53 | 0.641 | 3.6e-10 | 236 | 412 |
| UHRF1 | 0.641 | 6.1e-05 | 1360 | 960 |
| MAPKAPK2 | 0.626 | 3.3e-08 | 388 | 488 |
| CREBBP | 0.624 | 4.2e-04 | 351 | 408 |
| PAK2 | 0.616 | 1.3e-03 | 838 | 673 |
| MAPK14 | 0.612 | 6.3e-07 | 297 | 345 |
| NR4A1 | 0.61 | 1.1e-06 | 58 | 73 |
| SPP1 | 0.609 | 1.7e-03 | 1426 | 2958 |
| CCNB1 | 0.605 | 3e-03 | 358 | 637 |

**Note:**

AUC < 0.6: effect is small for clinical utility.

AUC 0.6-0.7: a cancer biomarker with potential clinical utility.

AUC 0.7-0.8: top quality cancer biomarker.
